# Supramolecular gels with high strength by tuning of calix[4]arene-derived networks

**DOI:** 10.1038/ncomms7650

**Published:** 2015-03-23

**Authors:** Ji Ha Lee, Jaehyeon Park, Jin-Woo Park, Hyo-Jun Ahn, Justyn Jaworski, Jong Hwa Jung

**Affiliations:** 1 Department of Chemistry and Research Institute of Natural Sciences Gyeongsang National University, Jinju 660-701, Korea; 2 School of Materials Science and Engineering, Gyeongsang National University, Jinju 660-701, Korea; 3 Department of Chemical Engineering, Hanyang University, Seoul 133-791, Korea; 4 Institute of Nanoscience and Technology, 222 Wangsimni-ro, Seoul 133-791, Korea

## Abstract

Supramolecular gels comprised of low-molecular-weight gelators are generally regarded as mechanically weak and unable to support formation of free-standing structures, hence, their practical use with applied loads has been limited. Here, we reveal a technique for *in situ* generation of high tensile strength supramolecular hydrogels derived from low-molecular-weight gelators. By controlling the concentration of hydrochloric acid during hydrazone formation between calix-[4]arene-based gelator precursors, we tune the mechanical and ductile properties of the resulting gel. Organogels formed without hydrochloric acid exhibit impressive tensile strengths, higher than 40 MPa, which is the strongest among self-assembled gels. Hydrogels, prepared by solvent exchange of organogels in water, show 7,000- to 10,000-fold enhanced mechanical properties because of further hydrazone formation. This method of molding also allows the gels to retain shape after processing, and furthermore, we find organogels when prepared as gel electrolytes for lithium battery applications to have good ionic conductivity.

## Abstract (web summary)

The physical properties of gel materials makes them attractive options in various applications, but supramolecular gels typically lack mechanical strength. Here, the authors present a calix[4]arene-based supramoleculer gel tuned to possess high tensile strength.

## Graphical abstract

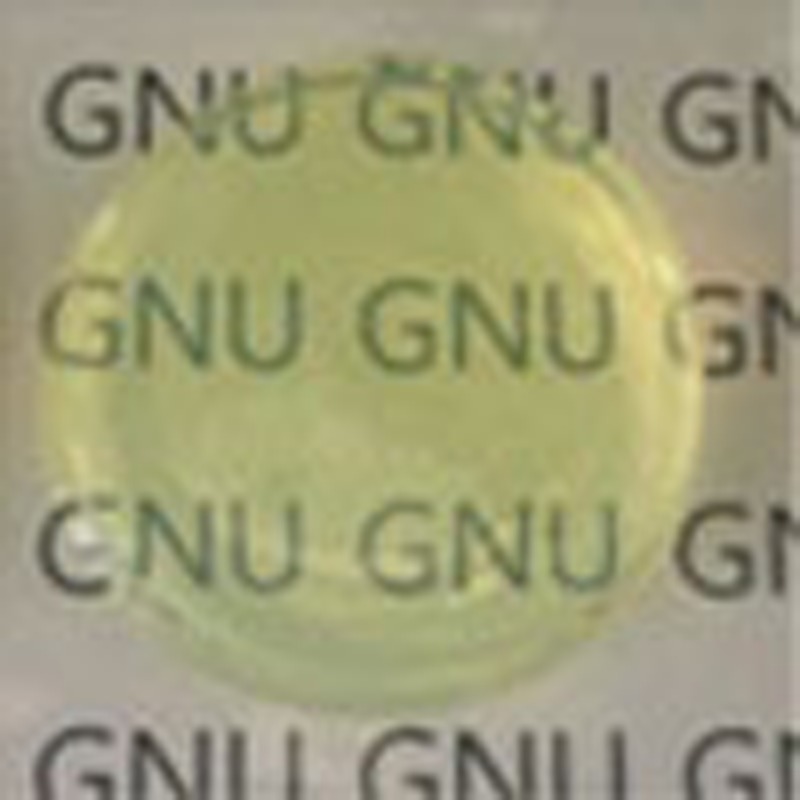

Soft material development has seen a wealth of applications for viscoelastic systems such as organogels and hydrogels, particularly for areas including controlled release^1^ and soft tissue reconstruction^2^. Aside from biomedical applications, gel systems have been implemented in energy capture and storage^3^ as well as sensing areas^4^ by revealing exciting responsive properties to external stimuli such as temperature, pH, light and electric field^5^. In a typical gel system, a gelator component makes use of extended structures to facilitate the formation of thermodynamically stable networks via physical or chemical interactions. In contrast to most chemically formed gels that have irreversible shapes due to permanent network structures, physical gels offer reversible networks capable of functional responsiveness and interesting self-healing properties^6^. These extended network structures often utilize polymers or fibrous supramolecular assemblies, which in the case of a hydrogel are closely associated with a large proportion of water-molecules that remain within the networked structure. Similarly, in the case of an organogel, apolar solvent molecules are associated with the network depending on the properties of the component gelators implemented.

Traditionally, hydrogel and organogel systems derived from polymers have been used in application areas where their characteristically weak mechanical properties would not be a limitation. Recent works have looked to forming hydrogels using polymeric composites with clay^7^,^8^,^9^, polymers reinforced with silica^10^ or peroxidized microspheres^11^, and mechanophores capable of shear force activated crosslinking^12^. Despite improved compressive strengths, in general the tensile strength remained largely unaffected. By employing a secondary phase of embedded microgel particles, a striking enhancement in tensile strength was observed by the group of Jian Ping Gong in polymeric hydrogels^13^; although, network rupture in the microgel components was found to cause irreversible gel damage. The same group later reported that polymers exhibiting a mixture of cationic and anionic repeats could provide a mixture of strong and weak bonds allowing ductile hydrogels to have tunable tensile strength up to 2 MPa (ref. 14). Recently, polyurethane-urea-derived hydrogels have moved past this boundary by demonstrating controllable tensile strength from 3.3 to 34 MPa depending on the diisocyanate content^15^. Although such advances provide a bright outlook for implementation of polymeric hydrogel systems in high strength application areas, similar advances have yet to be made for conventional supramolecular hydrogel systems.

Compared with conventional polymeric gelator systems, supramolecular gels such as hydrogels derived from cyclodextrin-based gelators^16^ have yet to yield mechanically robust materials for applications requiring high strength. In one example, cyclodextrin-derived supramolecular hydrogels revealed tensile strengths of 18 kPa; however, this system instead provided an impressive reversible elasticity up to 180% strain^17^. For many supramolecular gels, the inherently weak nature of non-covalent interactions that comprise the self-assembled network structures may cause such low-mechanical strengths. Attempts to overcome this limitation by strengthening supramolecular hydrogels using multivalent interactions have had some success. In a specific example, researchers developed hydrogels from zwitterionic amphiphiles encompassing an amino-acid head group and glycolipid hydrogelator^18^. Such a molecular gelator that can allow multiple orthogonal binding sites, may potentially yield a ‘cluster effect’ wherein complexes exhibit increased stability^19^ that may translate to enhanced mechanical properties. Although overcoming some of the mechanical fragility associated with supramolecular gels, the mechanical properties of the aforementioned hydrogel were nevertheless still only on par with that of agarose-based gels.

Aside from the mechanical limitation put forth, supramolecular hydrogels derived from low-molecular-weight gelators may face additional issues related to solubility in water, as many macrocyclic gelators are predominantly hydrophobic. Reports using calixarene-based hydrogels, although rare, provide evidence that proper functionalization of these macrocycles can yield effective hydrogelators^20^,^21^,^22^,^23^; however, we could not find any reports on their mechanical properties. The scarcity of studies on the mechanical properties of organogels and hydrogels prepared from self-assembled low-molecular-weight gelators may stem from these materials being very weak.

Herein we reveal an alternative strategy for generating calix[4]arene-derived hydrogels with high-mechanical strength for a supramolecular gel. The tensile strengths of the hydrogels approached 25 MPa (and greater than 40 MPa in the case of the organogel), which surpasses the mechanical properties of many polymer-based physical gels. In this system, we circumvent solubility issues by first generating an organogel from a calix[4]arene-derived gelator in dimethyl sulfoxide (DMSO) and subsequently utilizing solvent exchange after gel formation for *in situ* hydrogel formation. The sensitivity of the gelator components to the conditions of the hydrazone reaction allows the effective tuning of not only the conversion pathway of the gelator but also the network structure to provide a controlled means of strengthening the hydrogels mechanical properties. The material is ductile and offers retention in shape, even after solvent exchange; which makes this strategy using a monomeric gelator system effective for molding of freely-standing objects. When implementing the organogels as gel electrolytes, we observe good ionic conductivities. In addition, we find the extracted liquid from the processed hydrogels to be non-toxic under *in vitro* human cell culture conditions. As we will discuss, this new material offers the potential to extend the possible applications of supramolecular gel systems to more mechanically demanding roles.

## Results

### Supramolecular organogels formed by oligomerized gelators

**Figure 1:**
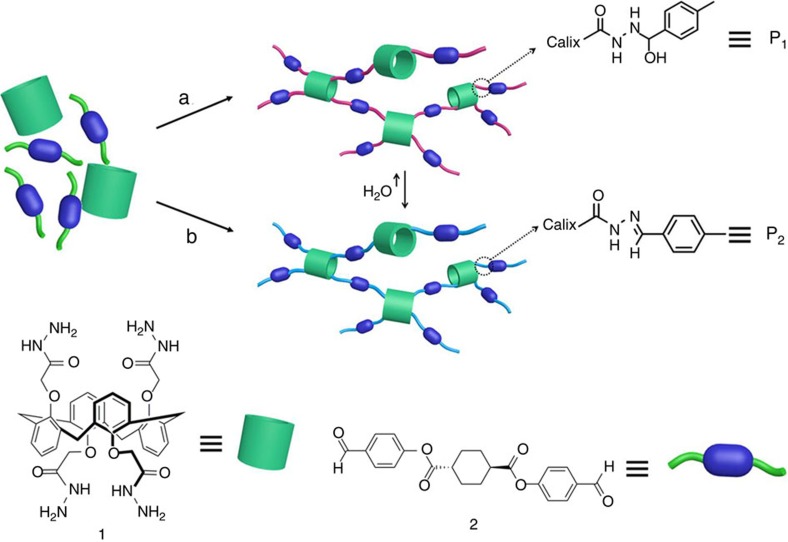
Schematic of gel formation by hydrazone reaction. The formation of oligomers from calix[4]arene-based gelator precursors by hydrazone reaction (a) without addition of acid and (b) with addition of acid.

To provide a gelator that could be formed *in situ*, we first synthesized calix[4]arene-derivative (**1**) and diphenyl terephthalate-derivative (**2**) precursors possessing reactive hydrazide and aldehyde groups, respectively. The hydrazide moieties of precursor **1** upon reaction with the aldehyde groups of precursor **2** result in hydrazone formation, wherein these linkages can offer amenable hydrogen-bonding sites to facilitate non-covalent assembly. The resulting gelator consists of 1,3 alternate-con-type calix[4]arene as the core component bordered by hydrazone groups linking with the hydrophobic diphenyl terephthalate derivative (Fig. 1 and Supplementary Fig. 1). Subsequent oligomerization can furthermore provide a larger network-structured gelator that is inherently less susceptible to crystallization. Importantly, this can be carried out *in situ* by the formation of hydrazone bonds between any tetrahydrazide-bearing **1** and up to four molecules of the aldehyde functionalized **2**. The linking of gelator precursors through hydrazone bonds yields a short oligomer network (Fig. 1) that can be controlled by addition of different catalytic amounts of acid. The hydrogen-bonding capabilities of the oligomeric gelator components provide a framework for inter-gelator hydrogen bonding into an extended network for supramolecular gel formation.

### Effects of hydrazone formation rate on organogel properties

**Figure 2:**
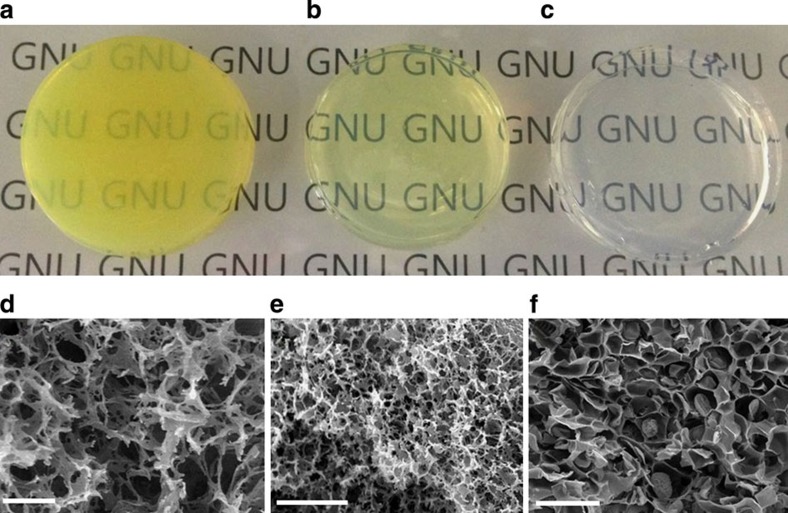
Photograph and SEM images of organogels at different concentration of HCl. (**a**) Organogel-5 and (**d**) SEM of corresponding xerogel (scale bar, 2 μm) formed using 10 nmol HCl (after 24 h reaction), (**b**) organogel-6 and (**e**) SEM of corresponding xerogel (scale bar, 2 μm) formed using 5 nmol HCl (after 24 h reaction), as well as (**c**) organogel-7 and (**f**) SEM of corresponding xerogel (scale bar, 50 μm) formed without addition of HCl (after 36 h reaction) were all produced using a mixture of **1** (42 mmol) and **2** (84 mmol) in DMSO.

To observe the effect on gel formation, HCl was systematically added to solutions containing **1** and **2** in DMSO (3wt% with respect to **1**) at a 1:2 molar ratio to provide equivalent proportions of hydrazide and aldehyde functional groups. Transparent organogel-7 (generated in the absence of acid) increased viscosity after 1 day, forming a weak gel capable of handling 1.5 days after initiating the reaction (Supplementary Fig. 2). In contrast, increasing the amount of HCl in the solutions to 10 nmol (organogel-5) and 5 nmol (organogel-6) resulted in the rapid onset of gel formation by 11 and 15 min, respectively (Supplementary Figs 3 and 4). Although the addition of HCl clearly accelerated formation of oligomerization in the organogels, it was also found to elicit a yellow hue, believed to result from carbazone formation^24^,^25^, and also the appearance of turbidity in the resulting gels (Fig. 2). Longer reaction times and higher HCl content resulted in an increased yellow hue for the gels. Upon measuring the sample absorbance at 450 nm (Supplementary Fig. 5), almost no loss in transparency was observed, even after several days, for the organogel prepared in the absence of acid, referred to herein as organogel-7.

In contrast, significant turbidity was observed shortly after gel formation when gel formation was conducted in the presence of 10 nmol HCl (organogel-5) and 5 nmol HCl (organogel-6). We suspected that the turbidity was strongly related to the structural formation within the gel, hence, we observed the respective organogels network morphology by scanning electron microscope (SEM; Fig. 2). Freeze-dried organogels formed with different amounts of HCl showed very distinct microstructured networks as revealed by SEM. Although organogel-7 revealed a honeycomb-like structure with large open plate-like networks (Fig. 2f), the ogranogel-6 and organogel-5 samples had comparatively much smaller pore structures and their networks appeared more like heterogeneous fibrils (Fig. 2d,e). Despite the heterogeneity for the acidic conditions, the micropores still appear to be highly interconnected.

In an effort to follow changes in the molecular structure of the gelator associated with oligomerization during organogel formation, the evolution of the chemical species over time with respect to different catalytic amounts of HCl was determined by nuclear magnetic resonance spectroscopy (NMR). Measurements of the ^1^H NMR spectra at various points during gelation allowed the extent of hydrazone bond formation to be evaluated from the ratio of the aldehyde proton of precursor **2** (peak 7) to the –NH proton peak present in the gelator product after coupling (Supplementary Figs 6–8). The ratio of the integrals of peak 12 and peak 7 are increasing in the spectra (Supplementary Fig. 6a–d) because of the formation of the hydrazone group as the reaction proceeds. The integral ratio of peak 12 to peak 7 (Supplementary Fig. 6e–h) may seem to decrease because of broadening of the NMR peak resulting from strong gel formation when the hydrazone reaction reaches equilibrium as shown in Supplementary Fig. 9. From these data, the degrees of hydrazone linkage formation were estimated to reach 81%, 73% and 40% reaction for organogel-5, -6 and -7, respectively. In addition, the reactions were found to reach equilibrium (Supplementary Fig. 9) within 30 min for the 10 nmol condition of organogel-5 and within 60 min for the 5 nmol condition of organogel-6 thereby resulting in coupled gelators comprised of 4-mers and 3-mers, respectively, based on an average across samples. The gel formed without addition of acid (organogel-7), in contrast, required at least 60 h to reach equilibrium of the oligomerization reaction, resulting in gelators comprised of predominantly 4-mers.

To confirm that the hydrazone reaction did not continue to polymerize the gelator into a covalently cross-linked network structure, we verified the reversibility of gel formation. Repeated measurements of the sol-gel transition were examined using differential scanning calorimetry (DSC) to reveal endothermic reaction temperatures corresponding to a *T*_gel_ of 105±3 °C, 100±2 °C and 98±2 °C for organogel-5, -6 and -7, respectively (Supplementary Fig. 10). These sol-gel transition temperatures were reproducible over the three repeated observations of the organogels. The endothermic peaks occur at the transition of the organogel into a solution phase and have a relatively broadened thermogram in comparison to purely monomeric organogels, which is due to the occurrence of a distribution of multi-meric species arising from our hydrazone reaction. Thus, we can be certain the resulting supramolecular gel was formed from discrete gelator species that were multi-meric rather than extended polymer network structures. In addition, a tube inversion method with heating and cooling of organogel-5 provided further visual confirmation of the reversible sol-gel transition indicating these are supramolecular gels and offered supporting assessment of the sol-gel transition temperature (Supplementary Movie 1).

### Mechanical properties of organogels

**Figure 3:**
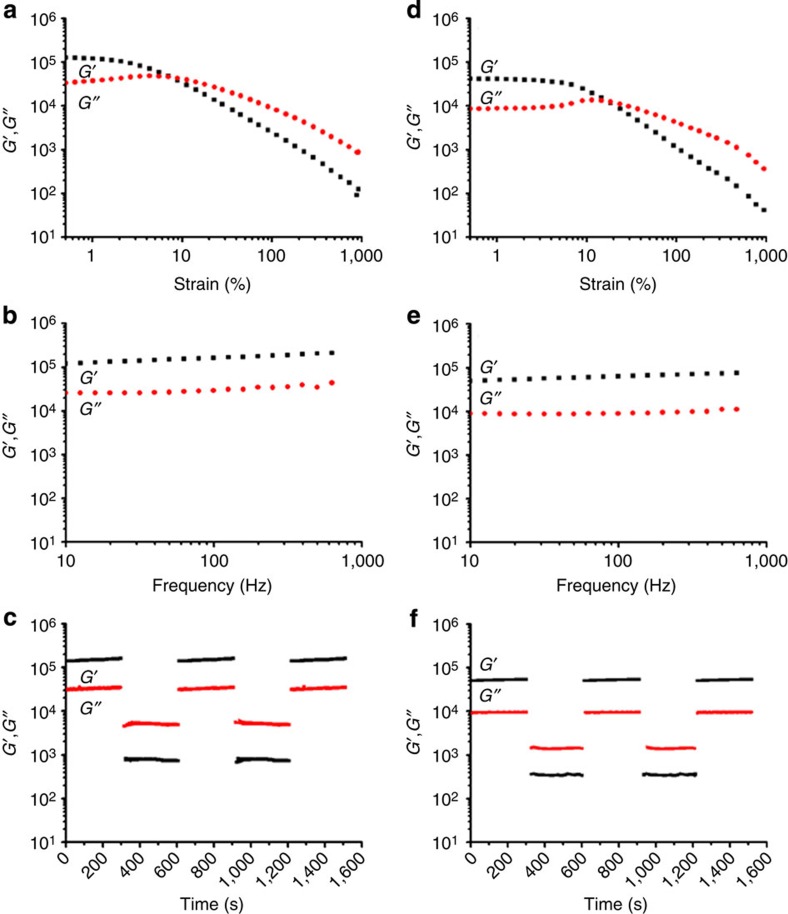
Rheological properties of organogels prepared by hydrazine reaction for 24 h. Storage (*G*′) and loss (*G*") values of organogel-5 (**a**–**c**), formed using 10 nmol HCl, and (organogel-6 (**d**–**f**), formed using 5 nmol HCl. Measurements of strain sweep tests at 0.5–1,000% for (**a**) organogel-5 and (**d**) organogel-6. Frequency sweep tests (from 10 to 1,000 Hz) for (**b**) organogel-5 and (**e**) organogel-6. Continuous step strain measurements at 0.5 and 1,000% for (**c**) organogel-5 and (**f**) organogel-6. All experiments conducted at 25 °C.

Having witnessed the organogels as being robust, we examined their mechanical properties in detail by first evaluating the storage (*G*′) and loss (*G*′′) moduli by rheometric measurement (Supplementary Movie 2). Strain sweep, frequency sweep and continuous step strain tests were conducted for each of the organogels, and the mechanical properties of organogels during evolution of gel formation as well as subsequent ageing were also examined (Fig. 3 and Supplementary Fig. 11). Among the different treatment conditions, organogel-5 showed the highest storage modulus as impacted by the increased rate of oligomerization under these conditions. Strain amplitude sweeps of the samples demonstrated an elastic response typical of gels (Fig. 3). The loss modulus of organogel-5 was found to rapidly decrease above the critical strain region (*γ*=20%), indicating a collapse of the gel state to a quasi-liquid state. After large-amplitude oscillatory break-down, the recovery of the rheological properties occurred quickly, and under the application of the large-amplitude oscillatory force (*γ*=1,000%; frequency, *ω*=6.0 rad s^−1^ (1.0 Hz)), we observe a decrease in the *G*′ value from 0.45 MPa to 0.03 KPa, resulting in a quasi-liquid state (tan *δ*=*G*′′/*G*′≈4.5). When the amplitude is decreased (*γ*=0.5%) at the same frequency (1.0 Hz), we see the *G*′ recovers quickly to the initial value and the system returns to a quasi-solid state (tan *δ*=*G*′′/*G*′≈0.21). Upon comparison with organogel-6, the *G*′ and *G*′′ values of organogel-5 were slightly higher than those observed for organogel-6 after reaching equilibrium.

At equilibrium, each of the organogels exhibited typical strain thinning responses owing to alignment of the microstructure; however, a weak strain overshoot could be observed when measurements were taken before complete equilibrium gel formation as was most pronounced in the case of organogel-7 at 36 h (1.5 day) after initiating the reaction (Supplementary Fig. 11). Such behaviour arises during the destruction and formation of network junctions, which may be expected to occur given the reaction does not yet reach equilibrium after 1.5 days^26^. For each of the organogels, we found the elastic response, *G*′, to dominate across all frequencies, and each of the organogels exhibited immediate recovery of *G*′ and *G*′′ in repeated step strain tests alternating above and below their critical strain amplitudes. The magnitude of the gels elastic response, *G*′, appeared to depend on the extent of acid-induced oligomerization; however, upon ageing, the gels were observed to have different behaviours. In looking more closely at the ageing behaviour of organogel-7, the dynamic modulus values gradually increased after initiating the reaction until the third day when the hydrazone reaction equilibrium had been reached. Even though the reaction had reached equilibrium by this point, further ageing continued to improve the rheological properties through stronger network formation, as we found after 1 week an increase in both the storage and loss moduli by two orders of magnitude (Supplementary Fig. 11). For each of the organogels, we could observe a quick recovery to their original shape once removing the applied load (Supplementary Movies 3 and 4).

**Figure 4:**
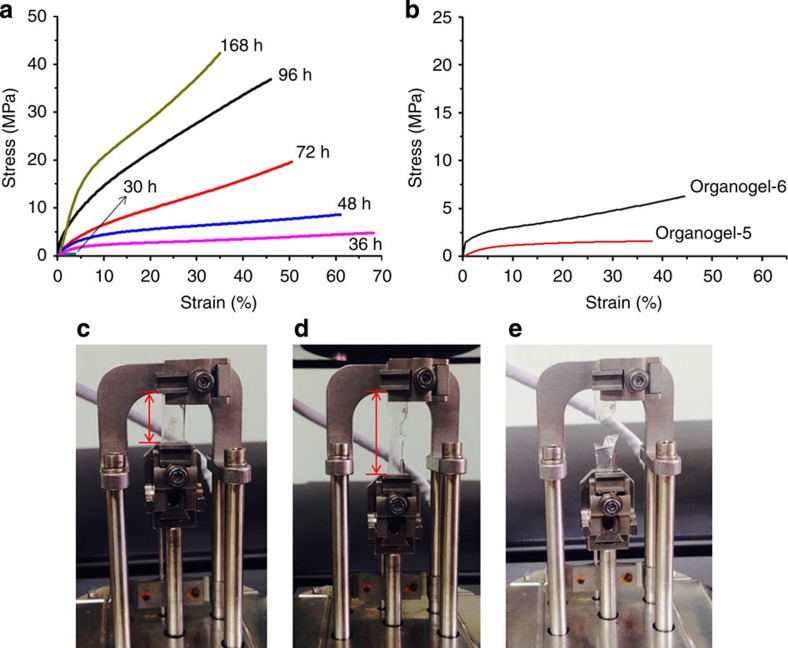
The engineering stress-strain tensile behaviour of organogels. (**a**) Tensile stress-strain curves of organogel-7 (having no HCl) prepared over (**a**) 30 h (light blue trace, designated by the black arrow), 36 h (1.5 days), 48 h (2 days), 72 h (3 days), 96 h (5 days) or 168 h (7 days) hydrazone reaction. (**b**) Tensile stress-strain curves of organogel-5 (red trace) and organogel-6 (black trace) prepared by hydrazone reaction for 24 h. Example of static tensile stress-strain test of organogel-7 after 36 h reaction illustrating (**c**) the initial state of the gel (scale bar, 2.5 cm), (**d**) the gel during pulling (scale bar, 4 cm), and (**e**) after mechanical failure of the gel.

To further confirm the interesting progression in the toughness and ductility with ageing, we examined the tensile stress-strain profiles of organogel-7 at additional time points throughout gel formation (Fig. 4a,c). After 1.5 days, the tensile strength of organogel-7 reached ~5 MPa (based on the engineering stress) with an almost 70% maximum strain, but upon continued ageing the gel strength exhibits a continued increase to ~20 MPa by day 3 and over 42 MPa by day 7, albeit with a simultaneous decrease in the maximum strain. This far exceeds the tensile strengths observed for organogel-5 and -6 and is one of the highest among any previously published supramolecular gel^14^,^20^,^21^,^22^,^27^, which is expectedly due to the strength of the calix[4]arene-based gelator network. The maximum strain of organogel-7 when examined with respect to reaction time over the course of 1 week reveals a gradual decline over time. Nonetheless, at longer reaction times, organogel-7 exhibited astounding tensile strengths, much higher than even many polymeric hydrogels such as those derived from poly(*N*-isopropylacrylamide)^28^ and polyampholytes^14^. This enhanced tensile strength of organogel-7 is due to the characteristics of the hydrazone reaction under non-acidic conditions, which produces –OH groups at the coupling site of precursors **1** and **2**. As a result, organogel-7 offers enhanced intermolecular hydrogen-bonding interactions in comparison to organogel-5 and -6 that have no auxiliary –OH groups and progressed at faster reaction rates relative to organogel-7. In the case of the stress-strain behaviour of organogel-5 and -6 after 24 h reaction (Fig. 4b), the organogel-6 was found to have a higher maximum strain and tensile strength compared with organogel-5. We find these observed maximum strains of our organogels surpass that of many hydrogels, even those composed of polyampholytes^14^. We may suspect that the improved ductility may in part arise due to more extensive connectivity in the network structure formed by the gelators when given sufficient time as was observed under slower reaction conditions.

### Hydrogel formation and characterization

Although the surprisingly high strength of organogel-7 is most certainly worthy of merit, because of solvent toxicity the application of this supramolecular gel to biomedical applications would be highly inappropriate. Therefore, we examined the formation of a hydrogel from organogel-7 by exchanging the DMSO solvent in the gel with H_2_O (thereafter referred to as hydrogel-7). As displayed in Supplementary Fig. 12, exchange of the solvent by 24 h immersion in water provided hydrogel-5, -6 and -7, which maintained their shape, although hydrogel-5 exhibited increased turbidity. After incubation in water, we found the DMSO was almost completely exchanged with H_2_O without losing the integrity of the gel network as determined by NMR spectroscopy (Supplementary Fig. 13).

It is important to note that such a hydrogel cannot directly form from precursors **1** and **2**; however, using this distinctive solvent exchange technique we can easily prepare a gelator from these precursors to generate an organogel that can thus undergo replacement with water for forming a functional hydrogel. Moreover, we found an increase in the storage and loss moduli after hydrogel formation (Supplementary Figs 14–17). After a hydrazone reaction time of 24 h for organogel-5 and -6 and 36 h for organogel-7, the organogels were then immersed in water for 24 h to allow solvent exchange to realize the corresponding hydrogels. By weighing the original organogels before and after solvent exchange with water and also after drying, we could determine the solvent content of the gels. Specifically, the water content was determined to be on the order of 93–95% of the total weight of the hydrogel (Supplementary Fig. 18 and Supplementary Movie 5). Significantly larger storage and loss moduli were observed in the resulting hydrogels compared with that of the original organogels after the corresponding hydrazone formation reactions. Hydrogel-7 exhibited the largest increase in elastic and viscous properties compared with the original organogel-7 from which it was produced (7.000- to 10,000-fold; Supplementary Figs 16 and 17). The rheological properties of this hydrogel-7 were almost the same as those found in the samples of organogel-7 aged for 1 week. Hydrogel-6 also had increased elastic and viscous properties and, to a similar extent, so did hydrogel-5. For hydrogels prepared from acidic conditions (hydrogel-5 and -6), we expect the addition of water acted to further catalyze the formation of hydrazone linkages to the enol form (similar to that of oligomers formed under non-acidic conditions as noted in Fig. 1, P_1_), thereby increasing the elastic properties via entangled gelator networks formed from extended oligomers. In addition, an increase in intermolecular hydrogen bonding could result from –OH formation as was noted for the reactions conducted in the absence of acid that were described above. This may be justified as the hydrogels exhibited more of a quasi-solid state as compared with that of the organogels, with the exception of organogel-7 and hydrogel-7, which gave similar rheological measurements.

To observe the sol-gel transition temperatures of the hydrogels, we carried out three repeated measurements by DSC (Supplementary Fig. 19), which indicated an increase in *T*_gel_ compared with that of the organogels. Owing to the possibility of water affecting the heat flow signal, we used a tube inversion method with heating and cooling of the gels to confirm the sol-gel transition temperature (Supplementary Movie 6). The reversibility of the sol-gel transition of the hydrogels can also be observed in the Supplementary Movie 6. To ensure that the DSC peaks arose from the sol-gel transition, we examined the powder XRD pattern to ensure it was not attributed to melting of any crystalline domains within the gel samples (Supplementary Fig. 20). We found that the various organogels and hydrogels each revealed only a single broad peak at 12–26°. This suggests that the gels are amorphous; hence, we could associate our observed DSC peak with the sol-gel transition. In existing literature^29^,^30^,^31^,^32^, the occurrence of X-ray scattering patterns can often be seen at small angles for organogels and hydrogels that possess lamellar structures when the small molecular gelators exist in well-ordered arrangements. In our case, however, the fairly large size of the 3- and 4-mers produced from the hydrazone reaction between **1** and **2** did not yield any sharp peaks in the powder XRD pattern indicating a disordered arrangement. As was the case for the original organogel-7, we also found that enhanced tensile strengths of the hydrogels occurred with an associated decrease in the maximum strain. It may be the case that the increased intermolecular hydrogen bonding or the extent of gelator entanglement could be the basis for the observed decrease in maximum strain.

In addition to rheological examination, we also measured the tensile stress-strain curves of the hydrogels prepared by exchange with water (Supplementary Fig. 21). The tensile strengths and maximum strains of both hydrogel-5 and hydrogel-6 were significantly enhanced compared with that of the original organogel-5 and -6. The increased tensile strengths of both hydrogel-5 and hydrogel-6 occurred to a greater extent than did the increase in maximum strain, which may be characteristically attributed to the entanglement between gel fibres provided in a chemical cross-linked structure. In contrast, the stress-strain behaviour of hydrogel-7 was decreased in terms of the maximum strain relative to the original organogel-7 after 36 h hydrazone reaction. This result indicates that hydrogel-7 became more brittle perhaps resulting from cross-linking within the structure.

With the aim of investigating the molecular level source of the hydrogel’s enhanced mechanical properties, we examined the NMR spectra of the organogels before and after the addition of water (Supplementary Fig. 22). As we could expect, the aldehyde proton peak decreased after addition of water. More importantly, the -OH proton ratios of organogel-5 and -6 after addition of H_2_O increased by approximately 1.4- to 1.5-fold relative to those of organogel-5 and -6 before addition of H_2_O, as attributed to water serving as catalyst. In contrast, organogel-7, which had similar mechanical properties before and after addition of H_2_O, showed no significant change in either their NMR spectra or Fourier transform infrared (FT-IR) spectra. The FT-IR spectra of xerogels produced by freeze drying of hydrogel-5 and -6, on the other hand (Supplementary Fig. 23), showed changes in their –OH vibrational peaks around 3,400 cm^−1^, which may indicate production of –OH during the hydrazone reaction without HCl. This supports the notion that additional intermolecular interactions may be occurring through hydrogen bonding of the extended gelators to provide enhanced tensile strength. As such, there would seem to be two different reaction pathways present depending on the presence of acid in the gel system.

Another fundamental transformation was discovered by SEM after hydrogel formation, which may offer an explanation for the strong mechanical properties. Specifically, we found the network structure of the organogel to be completely transformed into a lamellar structure after hydrogel formation in water as seen in the SEM of the corresponding freeze-dried xerogels (Supplementary Fig. 24). From the perspective of the multi-meric gelator fibres, a favourable realignment of the gel fibre could occurs through increased intermolecular hydrogen bonding as a result of the additional –OH groups formed upon immersion in water (Fig. 1). It remains to be seen if this change in microstructure may alternatively be attributed to unfavourable interaction parameters between the core calix[4]arene domain (**1**), the solvent, and the –OH bearing coupling domain (**2**). Prior works on gels comprised of hydrazone bonds have alluded to their aggregation as imposed by hydrogen bonding^27^ and the possibility of their dynamic nature allowing reversible covalent recombination^33^. The realignment of the gelator fibres in the lamellar structure of the hydrogel provides a clear indication for improvements in tensile strength as compared with the network structure seen in the organogels. As outlined schematically in Supplementary Fig. 25, the building blocks derived from the coupling reaction of **1** and **2** during organogel formation provided intermolecular hydrogen-bonding interactions between –C=O and –NH groups on nearby fibres. However, the partial –C=N bond that resulted from the hydrazone reaction for organogel formation was transformed upon submersion in water to produced new –NH and –OH groups on the surface of fibres during hydrogel formation. The additional intermolecular hydrogen bonds between –NH, –C=O and –OH groups may thereby induce alignment of fibres, due to strong fibre–fibre coupling, and this alignment of fibres could result in the formation of lamellar sheet. Thus, the mechanical properties of hydrogels with lamellar structures would be expected to exhibit a significant enhancement.

In getting back to the question of applicability to biomedical devices, we examined the cytotoxicity of liquid extracted from the hydrogels by addition of the liquid to *in vitro* cultures of adherent human cells, specifically using HeLa cells (Supplementary Fig. 26). MTT (3-(4,5-dimethylthiazol-2-yl)-2,5-diphenyltetrazolium bromide) assays reveal a noticeable decrease in cell viability with increasing amount of extracted liquid from samples that still contained trace amounts of DMSO, which is to be expected as long-term exposure to even low concentration of DMSO are known to be cytotoxic. In contrast, after sufficient replacement of DMSO with water by repeated immersion of the hydrogel, the extracted liquid showed no cytotoxicity, wherein the cell viability was similar to that of addition of pure distilled water. Further evaluations will be carried out in the future to determine the potential *in vivo* use of this new supramolecular hydrogel material towards biomedical applications.

### Organogels for battery applications

**Figure 5:**
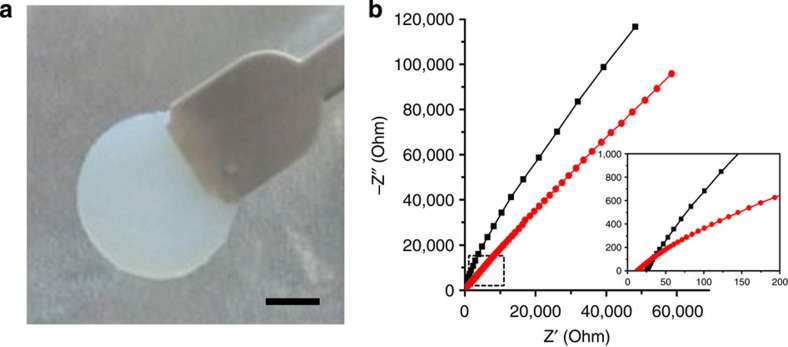
Photograph and electrochemical impedance spectroscopy of the gel electrolytes. (**a**) Photograph of a gel electrolyte disc (composite-II; scale bar, 2.5 mm) and (**b**) electrochemical impedance spectra of gel electrolytes prepared from two different composites of organogel-7. The inset provides an expanded view of the lower range measurements.

Examining other relevant areas for use of these unique gel systems, we prepared two different composite of organogel-7 as a gel electrolyte towards battery applications. Gel electrolyte discs were produced from organogel-7 with either LiNO_3_ (referred to as composite-I) or with a mixture of LiPF_6_ in EC/DEC (composite-II). Photographs of the composite gel electrolytes are shown in Supplementary Fig. 27, which reveal that the gel electrolyte can be produced as a freely standing and easily handled robust material. Although the composite-I gel electrolyte was colourless and transparent, we found the composite-II gel electrolyte to exhibit an opaque white colour. Using electrochemical impedance spectroscopy, the ionic conductivities of the gel electrolytes were estimated for each composite placed between a set of steel electrodes, and the corresponding Nyquist plot is displayed in Fig. 5. We find that the gel electrolyte composites showed good ionic conductivities of 8.05 × 10^−3^ and 4.29 × 10^−3^ S cm^−1^ for composite-II and composite-I, respectively. Although the conductivities were less than that of a corresponding liquid electrolyte, these conductivities were nonetheless on par with previously developed polymer gel type electrolytes^34^. The difference in ionic conductivity of composite-II (ca two times higher than that of composite-I) may be attributed to the mobility effects by the solvent^35^ and subtle variations in gel phase structure, as two different molding techniques were employed for these composites. Accordingly, further optimization of such supramolecular gel electrolytes may prove useful in future lithium battery applications, as bendable and flexible materials remain a desired design component for batteries used in wearable devices^36^.

In conclusion, ductile and high tensile strength hydrogels were produced by a robust new approach, which provides an alternative to supramolecular gel formation that have in the past been mechanically weak and unable to be molded into free standing objects^7^. According to our literature search, the work presented here is the first example of on-site supramolecular hydrogel formation via an organogel intermediate. Given the wealth of responsive and switchable gels that have been developed by incorporation of macrocycles, such as cyclodextrin among others^32^,^37^,^38^,^39^, the potential may exist for expanding our *in situ* hydrogel preparation method for the development of future smart-materials or dual-use functional/structural hydrogels by implementing calixarene-based gelators or related macrocyclic derivatives.

## Methods

### General characterization

Using a Bruker ARX 300, the ^1^H and ^13^C NMR spectra of the samples were obtained. A Shimadzu FT-IR 8400S was used to measure the FT-IR spectra in ATR mode over the range of 400–4,000 cm^−1^. A Hitachi U-2900 was used to determine the optical absorption spectra while a JEOL JMS-700 mass spectrometer was used to obtain the mass spectra. For DSC measurements, gel samples were hermetically sealed in a silver pan and referenced to a pan containing alumina. We employed a high-sensitivity differential scanning calorimeter (Hitachi DSC 7020) utilizing liquid N_2_ cooling unit with thermograms obtained at a 1.0 °C min^−1^ heating rate. Powder XRD patterns were measured on a Bruker AXS D8 DISCOVER.

### Preparation of supramolecular organogels and hydrogels

Compounds **1** and **2** were synthesized according to the detailed steps outlined in the Supplementary Methods (see Supplementary Figs 28–30 for full characterization). Compound **1** (15 mg, 42 mM) and compound **2** (16 mg, 84 mM) were first dissolved in DMSO (0.5 ml). Two samples (organogel-5 and -6) were then prepared in the presence of 10 and 5 nmole of HCl, respectively. Organogel-7 was prepared without addition of HCl. The gels were formed after simple mixing and incubation at room temperature. For preparation of hydrogel-5, -6 and -7, the corresponding organogels (3 wt% for **1**, 0.5 ml) previously prepared from DMSO (organogel-5 and -6 aged for 24 h; organogel-7 aged for 36 h) were immersed in H_2_O (50 ml) for 24 h at room temperature. After solvent exchange, the amounts of DMSO removed were evaluated by ^1^H NMR.

### Preparation of xerogels from organogels and hydrogels

Organogels and hydrogels were transferred into liquid N_2_ for 10 min and samples were then frozen at −40 °C overnight and freeze dried by vacuum at 0.1 Pa to yield the dry xerogels according to standard protocols^30^,^40^,^41^,^43^. The resulting xerogels were subsequently used for FT-IR, XRD and SEM measurements.

### Preparation of gel samples for rheological measurements

Compounds **1** (60 mg, 42 mmol) and **2** (64 mg, 84 mmol) were dissolved in 2 ml of DMSO with and without HCl in a 4.5-cm diameter round container. The reaction mixtures were maintained at a constant reaction temperature of 25 °C for a set period of time to allow gel formation. The organogels and hydrogels prepared by hydrazone reaction without or with subsequent solvent exchange were loaded onto the rheometer plate and then cut to exactly 4 cm diameter (equal to the 4 cm plate diameter) standard for measuring rheological properties.

### SEM observation

An FE-SEM, Philips XL30 S FEG field emission SEM was used to obtain images of the freeze-dried gel samples using an accelerating voltage 5–15 kV and an emission current of 10 μA. The observed gel samples were freeze dried to provide the corresponding xerogels and were observed from the side after cutting.

### Rheological properties

Rheological test of gels were carried out by using An AR-2000ex (TA Instruments Ltd) implemented with a 40-mm diameter parallel plate that was attached to a transducer. The gap in the setup for rheological testing of the gels was 1.0 mm and experiments were conducted at 25 °C. Strain sweep tests were performed with increasing amplitude oscillation up to 100% apparent strain on shear. Frequency sweeps were performed from 10–1,000 Hz. The recovery properties of the gels in response to applied shear force were investigated with the following 1,500 s procedure: 0.5% (300 s)→1,000% (300–600 s)→0.5% (600–900 s)→1,000% (900–1,200 s)→0.5% (1,200–1,500 s).

### Static tensile stress-strain test of organogels/hydrogels

Using a DMA Q800 (TA Instruments Ltd), we performed static mechanical testing on the gel samples under tensile loading at 25 °C. In our system, the tensile stress was considered to be the engineering stress as the force applied to the gel divided by the initial cross-sectional area of the gel, while the tensile strain was defined as the ratio of gauge length change of the gel relative to the initial gauge length. For static tensile stress-strain testing, gels with dimensions of 25 × 5 × 2 mm^3^ were used, and averages of the mechanical property tested were obtained over at least three specimens.

### Hydrazone reaction rate between 1 and 2

Compound **1** (42 mmol) was dissolved in DMSO-d_6_. Compound **2** (84 mmol) was added to the compound **1** solution. Next, a constant amount of DCl (0, 5 and 10 nmo1) was added to a given reaction mixture at room temperature. The rates for the hydrazone reaction between **1** and **2** with or without DCl were evaluated by ^1^H NMR. After a range of set reaction times, the rates for hydrazine reaction between **1** and **2** were evaluated. In addition, for a set reaction time upon addition of H_2_O (25 μl), we examined the ^1^H NMR spectra to investigate the influence of H_2_O on the hydrazone reaction.

### MTT cell viability assay

HeLa cells were cultured according to ATCC methods to a confluency of 50% before media replacement and addition of liquid test samples of DMSO, distilled water or liquid extracted from hydrogel-7 prepared samples. After 24 h of incubation, MTT reagent was added for 1 h at 37 °C. The assay/spectra acquisition were then carried out according to the manufacturer’s protocol (EZ-Cytox, Dail Lab Service).

### Preparation of gel electrolyte

Gel electrolytes were prepared through two different solution casting methods. In the first method, gelator precursors were dissolved in DMSO solvent as described above along with 0.5 M lithium nitrate (LiNO_3_) and stirring at room temperature for 1 h. The solution was next heated at 80 °C for 10 min. The transparent solutions were then cast into glass beakers and kept at room temperature overnight until the formations of the gel type electrolytes. Finally, the gel electrolyte was cut into circular discs with areas of 1.53 cm^2^. In the second method, gelator precursors were also added into DMSO solvent as described; however, they were first heated at 80 °C for 10 min before addition of Li. Once completely dissolved, a specific amount of 1 M LiPF_6_ in EC/DEC (1:1 (v/v)) was added to the gelator solution and the gel was cast in the same manner as described above to yield the gel electrolyte disc. All processes were conducted in an argon-filled glove box.

### Electrochemical measurement

The ionic conductivity of the gel polymer electrolytes were determined from the impedance spectrum using a blocking cell of which the electrolyte was sandwiched between two stainless steel electrodes in a Swagelok cell. Electrochemical impedance spectrum measurements were performed using a VMP3 (BioLogic) over a frequency range of 1 MHz to 100 mHz with a potentiostatic signal amplitude of 10 mV.

## Author contributions

J.H.L. contributed to the project design and performed the preparation of gel and mechanical properties and SEM images. J.P. performed the synthesis. J.-W.P. and H.-J.Ahn carried out the electrochemical measurement. J.H.J. and J.J. conceived the methodology and supervised the project. All authors edited the manuscript.

## Additional information

**How to cite this article:** Lee, J. H. *et al*. Supramolecular gels with high strength by tuning of calix[4]arene-derived networks. *Nat. Commun.* 6:6650 doi: 10.1038/ncomms7650 (2015).

## Supplementary Material

Supplementary Figures and Methods Supplementary Figures 1-30 and Supplementary Methods

Supplementary Movie 1 The sol-gel transition of organogel-5 prepared by aging for 24 hr

Supplementary Movie 2 Example of rheological measurement on organogel-6.

Supplementary Movie 3 Illustrative video showing (a) organogel-5 (left-side) by aging for 24 hr, (b) organogel-6 (center) by aging for 24 hr and (c) organogel-7 (right-side) by aging of 48 hr under compression by 1000g weight.

Supplementary Movie 4 Example of deformation and recovery of organogel-5 after 24 hr aging (right gel), organogel-7 after 36 hr aging (center gel), organogel-7 after 7 days aging (left gel).

Supplementary Movie 5 Illustrative video showing the high solvent content of hydrogel-6 by partial removal of solvent through squeezing of a gel sample.

Supplementary Movie 6 The sol-gel transition of hydrogel-6 prepared by solvent exchange.
