# Supplementary material for: Supramolecular gels with high strength by tuning of calix[4]arene-derived networks
Source: Nat Commun. 2015 Mar 23;6:6650. doi: 10.1038/ncomms7650 (PMC4383010; doi:10.1038/ncomms7650)
Supplement: Supplementary Figures and Methods — Supplementary Figures 1-30 and Supplementary Methods [file ncomms7650-s1.pdf]

Supplementary Figures

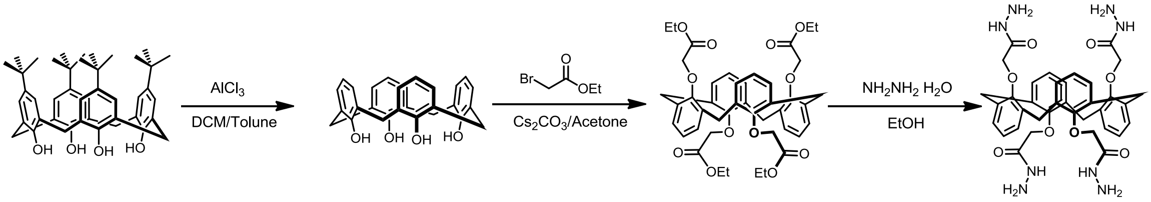

3 **4** **5** **1**

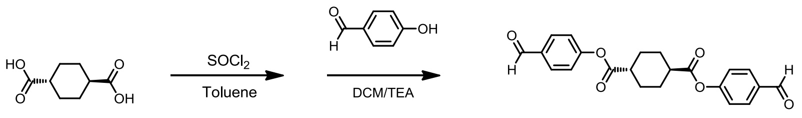

6 **2**

Supplementary Figure 1. Schematic of synthetic methods for compounds **1** and **2**.

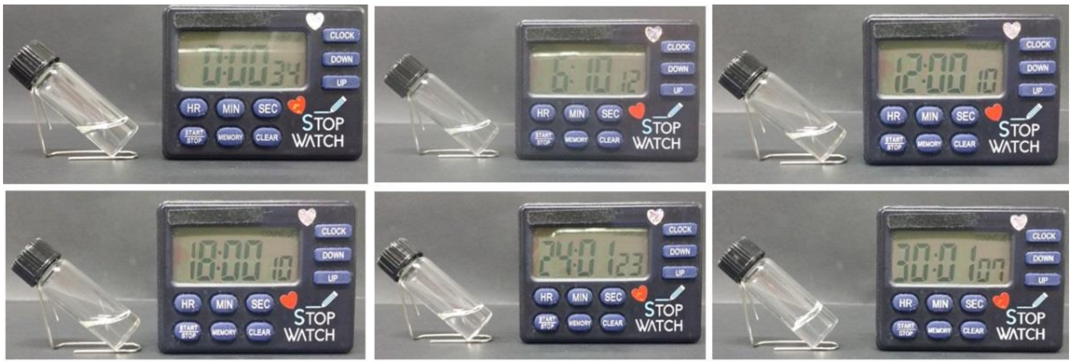

Supplementary Figure 2. The photograph for gel formation (orgnonogel-7) of **1**+**2** (**1**: 42 mM and **2**: 84 mM) in the absence of HCl by increasing hydrazone reaction times.

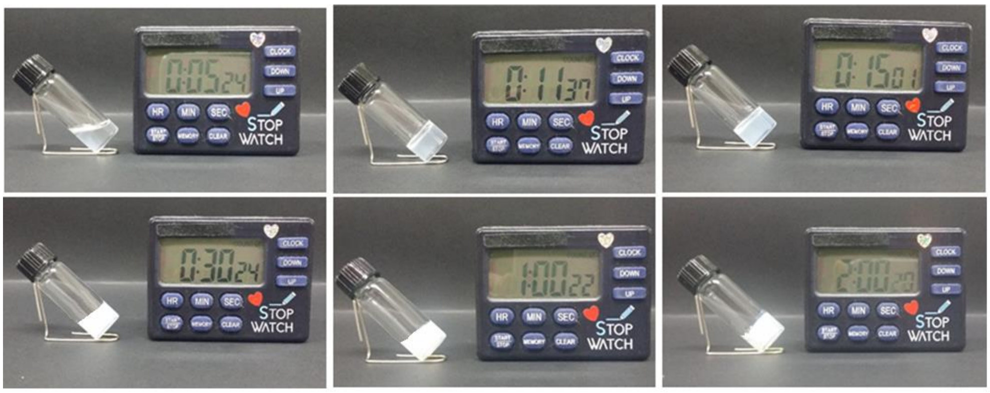

Supplementary Figure 3. The photograph for gel formation (organogel-5) of **1**+**2** (**1**: 42 mM and **2**: 84 mM) in the presence of HCl (10 nmol) by increasing hydrazone reaction times.

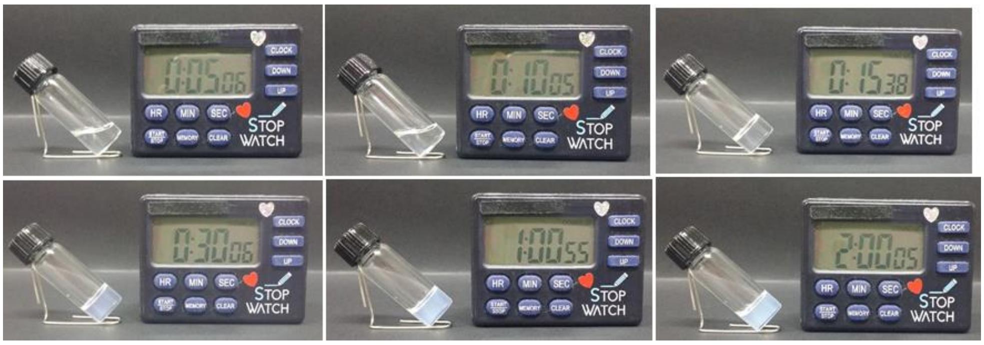

Supplementary Figure 4. The photograph for gel formation (organogel-6) of **1**+**2** (**1**: 42 mM and **2**: 84 mM) in the presence of HCl (5 nmol) by increasing hydrazone reaction times.

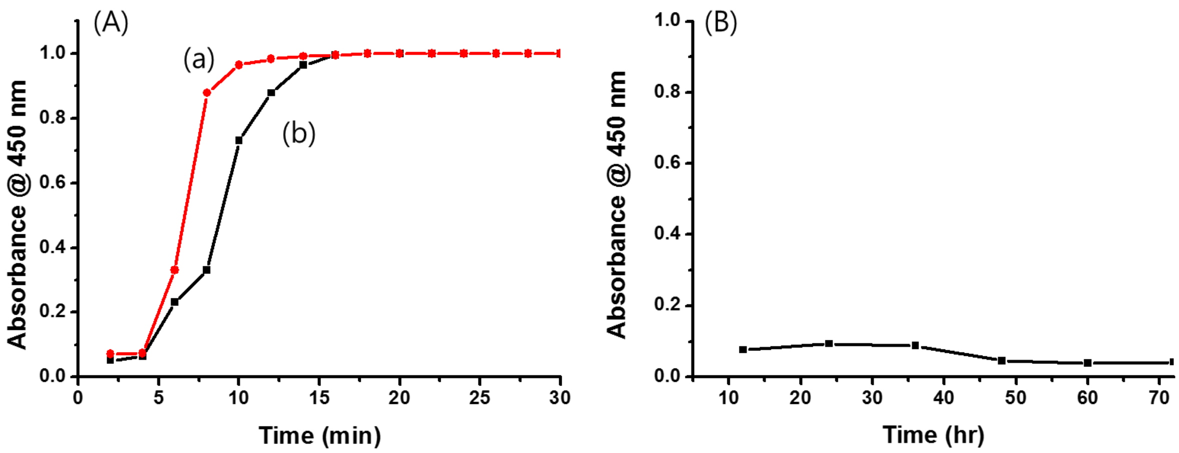

Supplementary Figure 5. Turbidity measurements show the time-dependent absorbance of 450 nm light of (A; a) organogel-5, (A; b) organogel-6 and (B) organogel-7.

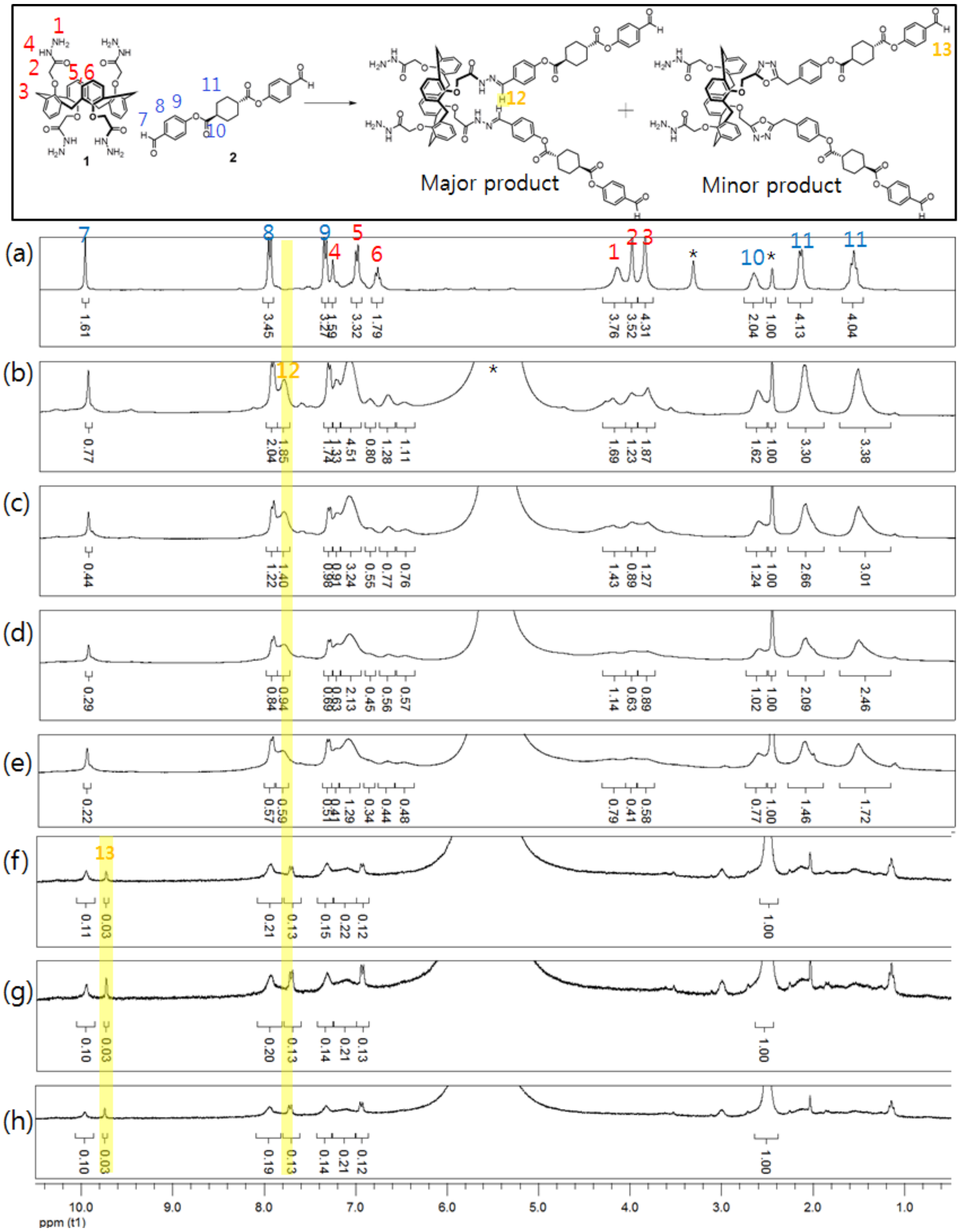

Supplementary Figure 6. NMR measurement of organogel-5 (3wt%, 42 mM for **1** and 84 mM for **2**) (a) before and after adding DCl (10 nmol), (b) 2 min, (c) 10 min, (d) 20 min, (e) 30 min, (f) 60 min, (g) 90 min and (h) 120 min in DMSO-d_6. Note the integrals from the aldehyde proton peak 7 relative to the generated NH peaks in the newly formed hydrazone group.

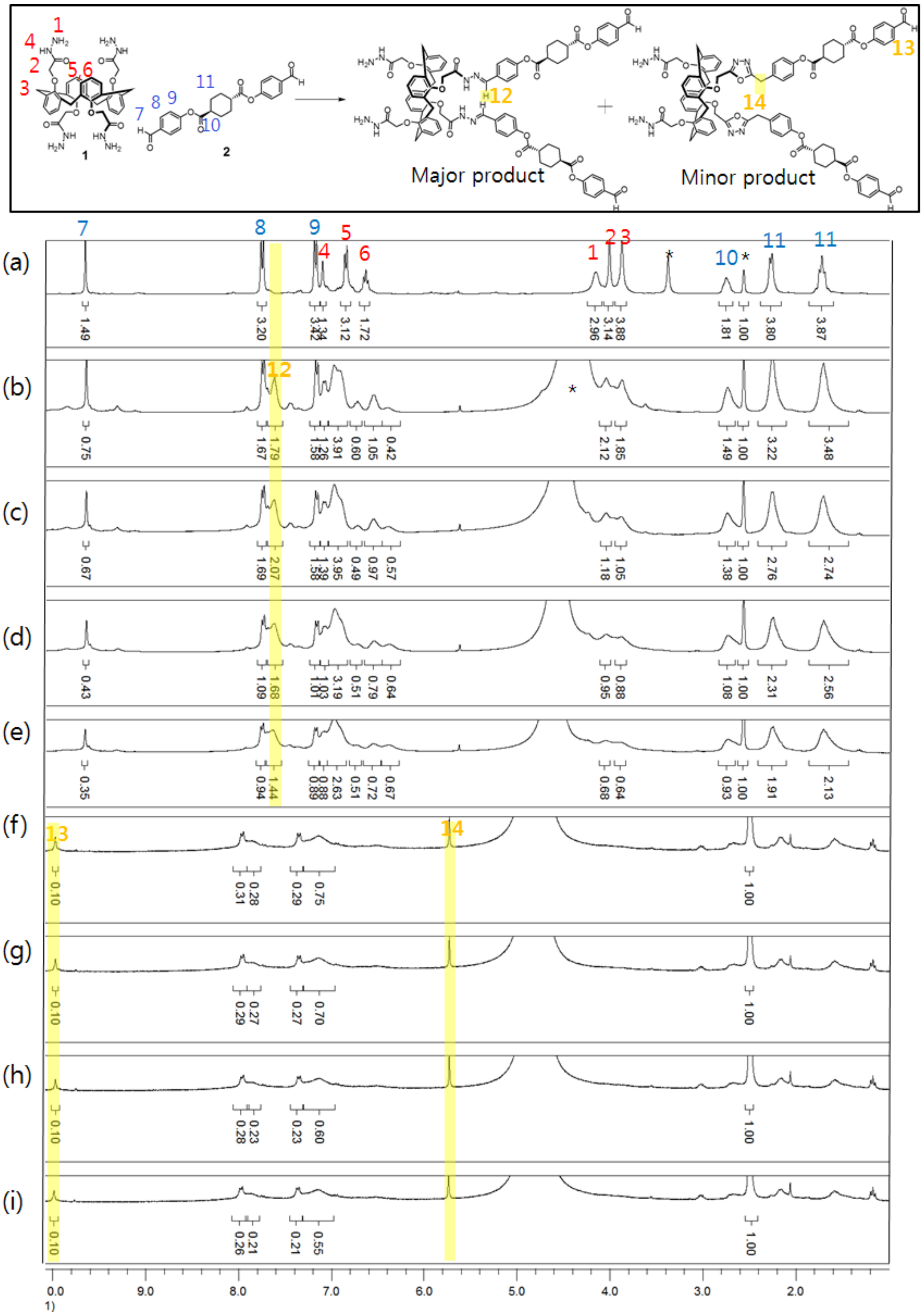

Supplementary Figure 7 NMR measurement of organogel-6 (42 mM for **1** and 84 mM for **2**) (a) before and after adding DCl (5 nmol), (b) 2 min, (c) 10 min, (d) 20 min, (e) 30 min, (f) 60 min, (g) 90 min, (h) 120 min and (i) 150 min in DMSO-d_6. Note the integrals from the aldehyde proton peak 7 relative to the generated NH peaks in the newly formed hydrazone group.

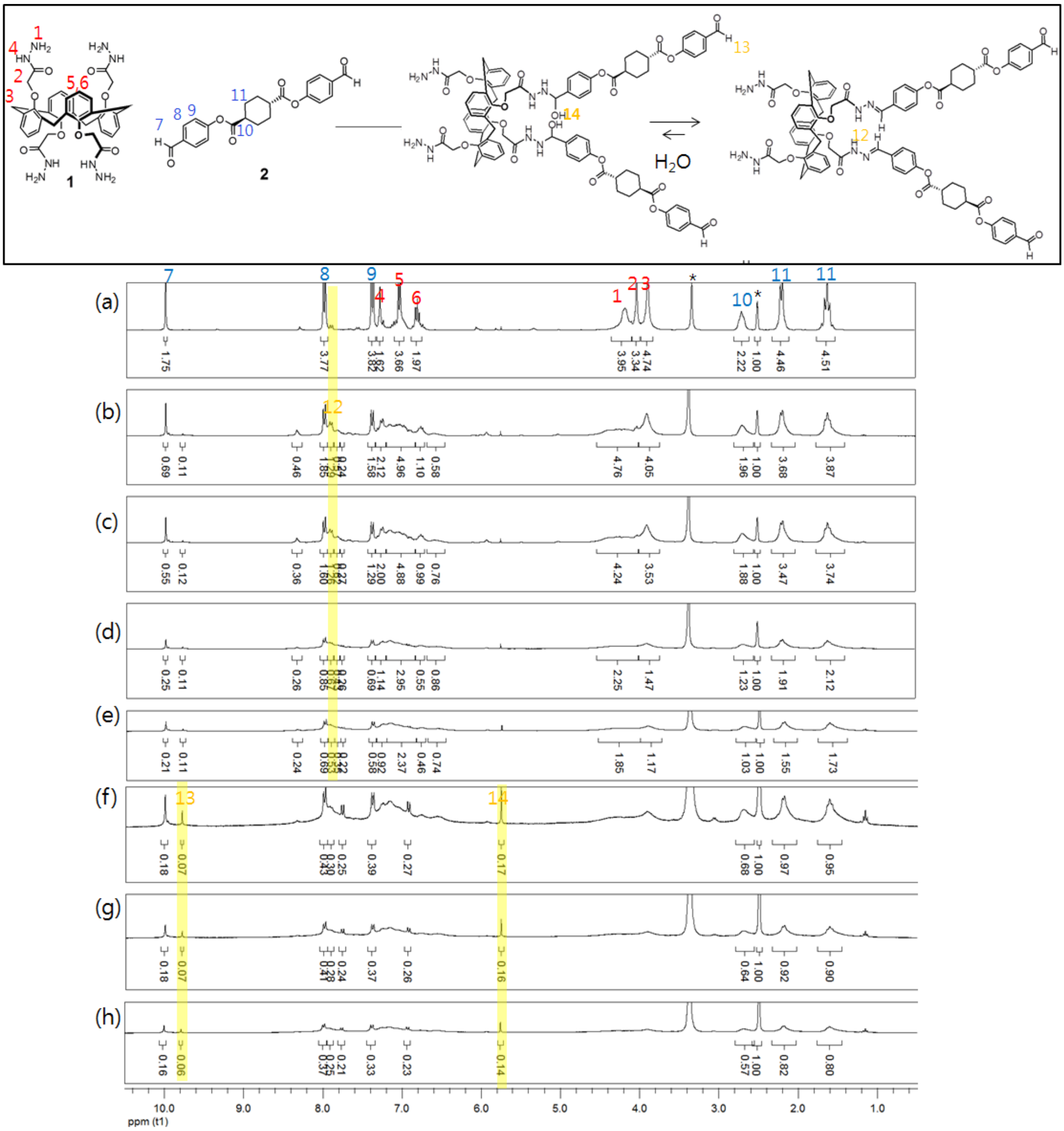

Supplementary Figure 8. NMR measurement of **organogel-7** (42 mM for **1** and 84 mM for **2**) without DCl after (a) 5 min, (b) 12 hr, (c) 18 hr, (d) 36 hr, (e) 42 hr, (f) 60 hr, (g) 68 hr and (h) 84 hr at pH 7 in DMSO-d_6. Note the integrals from the aldehyde proton peaks 7 relative to the generated NH peaks in the newly formed hydrazone group.

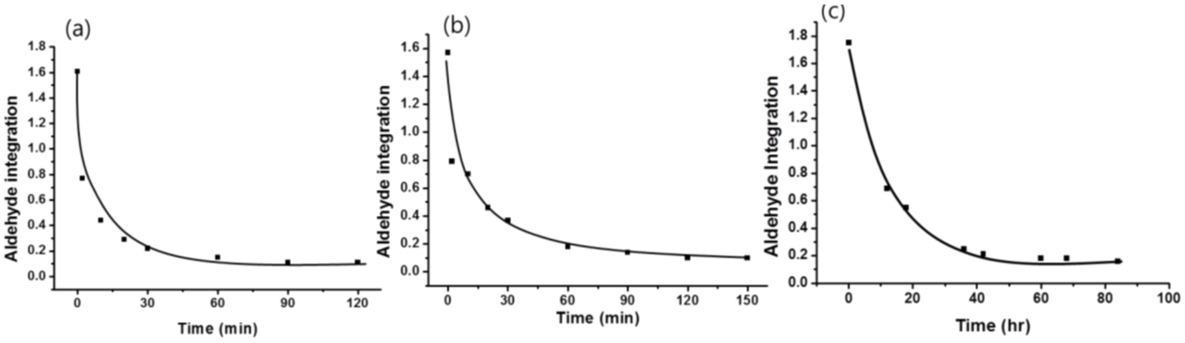

Supplementary Figure 9. The change in the amount of reactant (**2**) after product (hydrazone) formation as a function of time showing a plateau after equilibrium hydrazone reaction has been reached. The equilibrium hydrazone reaction can be seen to occur at different time points for (a) organogel-5, (b) organogel-6 and (c) organogel-7, respectively.

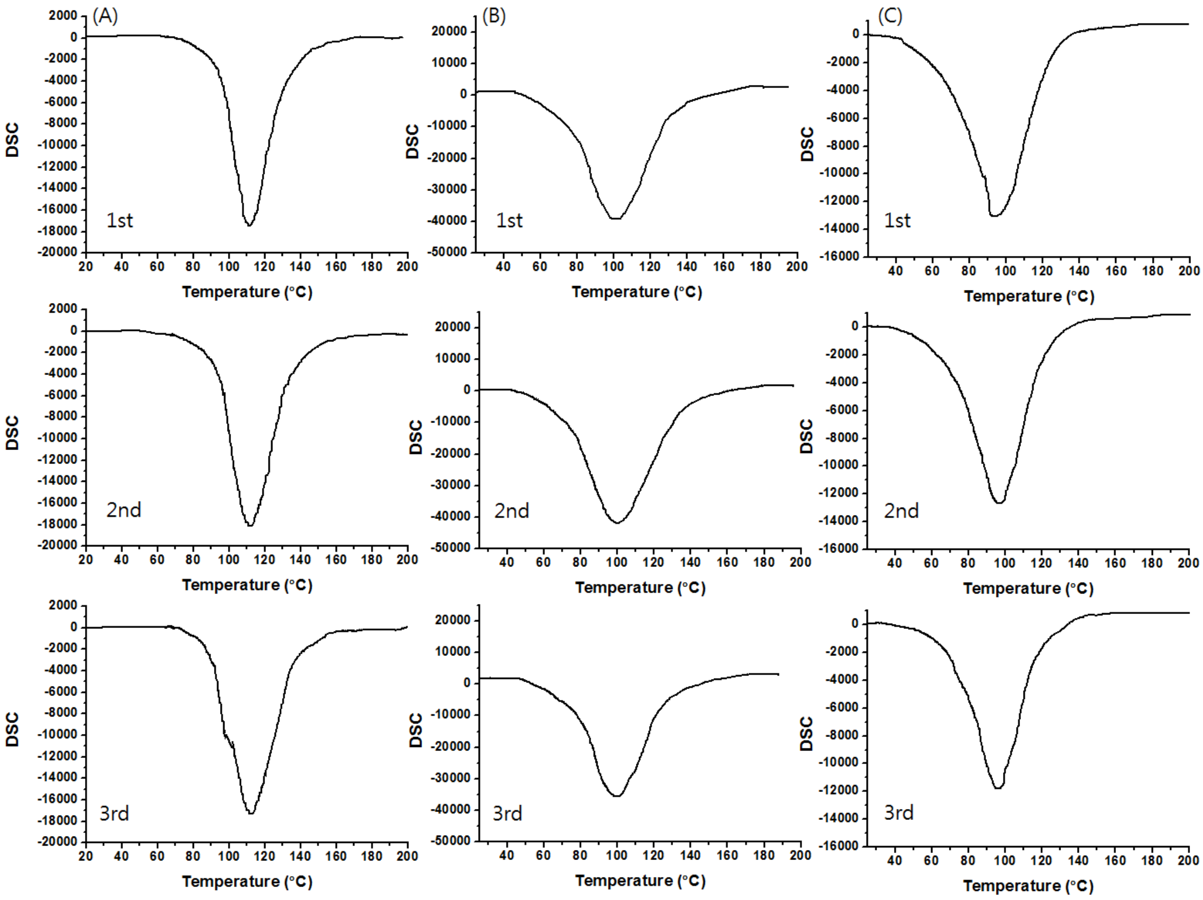

Supplementary Figure 10. Thermograms of DSC experiments for individual samples of (A) organogel-5 (24 hrs aging), (B) organogel-6 (24 hrs aging) and (C) organogel-7 (36 hrs aging). Each row represents subsequent measurements of the same individual samples taken after aging for an additional 24, 24, and 36 hrs for organogel-5, -6, and -7, respectively. To examine the gels after long aging times, each sample was examined under temperature ramping conditions rather than a temperature cycling program.

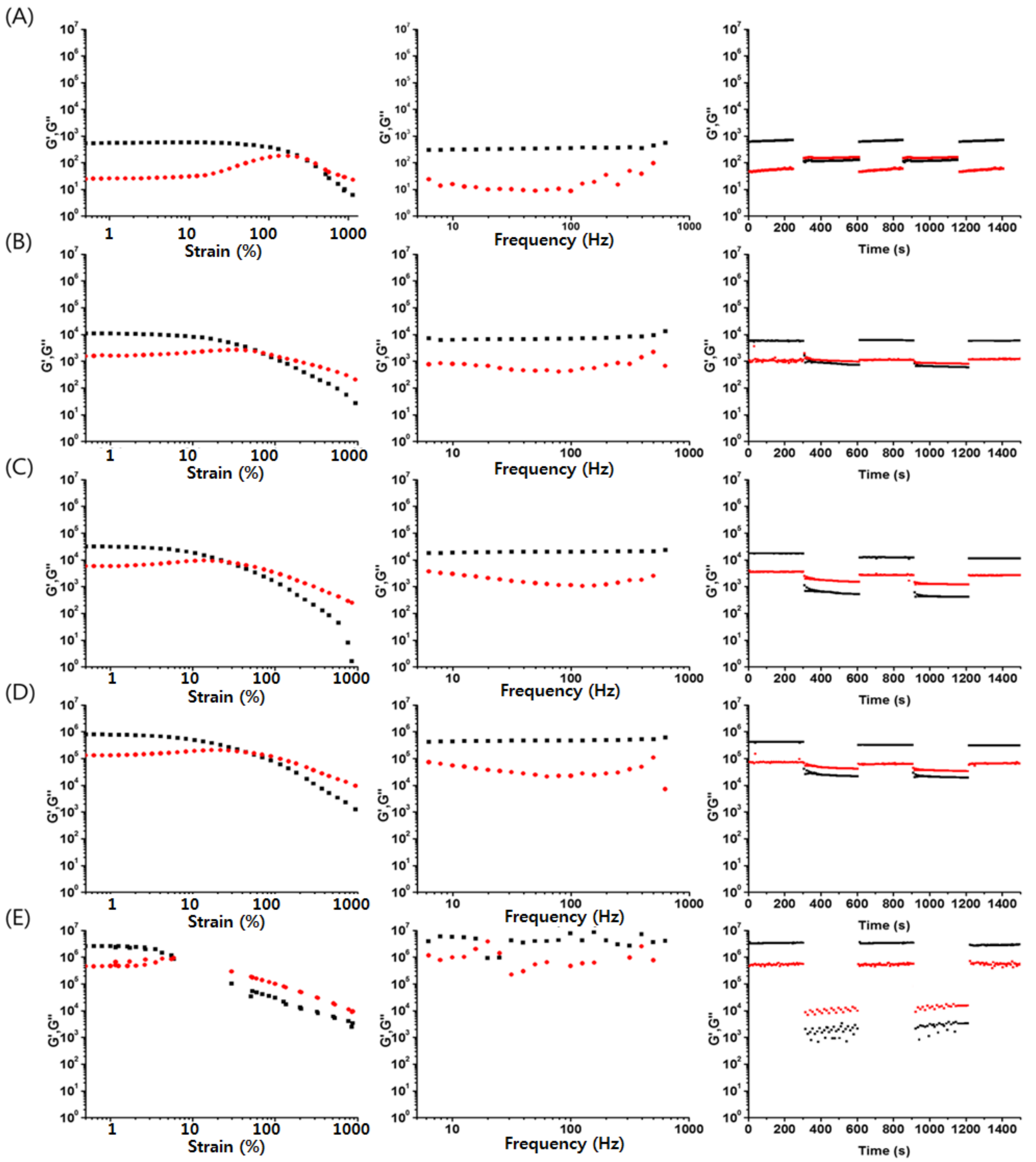

Supplementary Figure 11. Rheological properties (G’ black dot; G’’ red dot) of organogel-7 after aging for (A) 1.5 days, (B) 2 days, (C) 3 days, (D) 4 days and (E) 7 days.

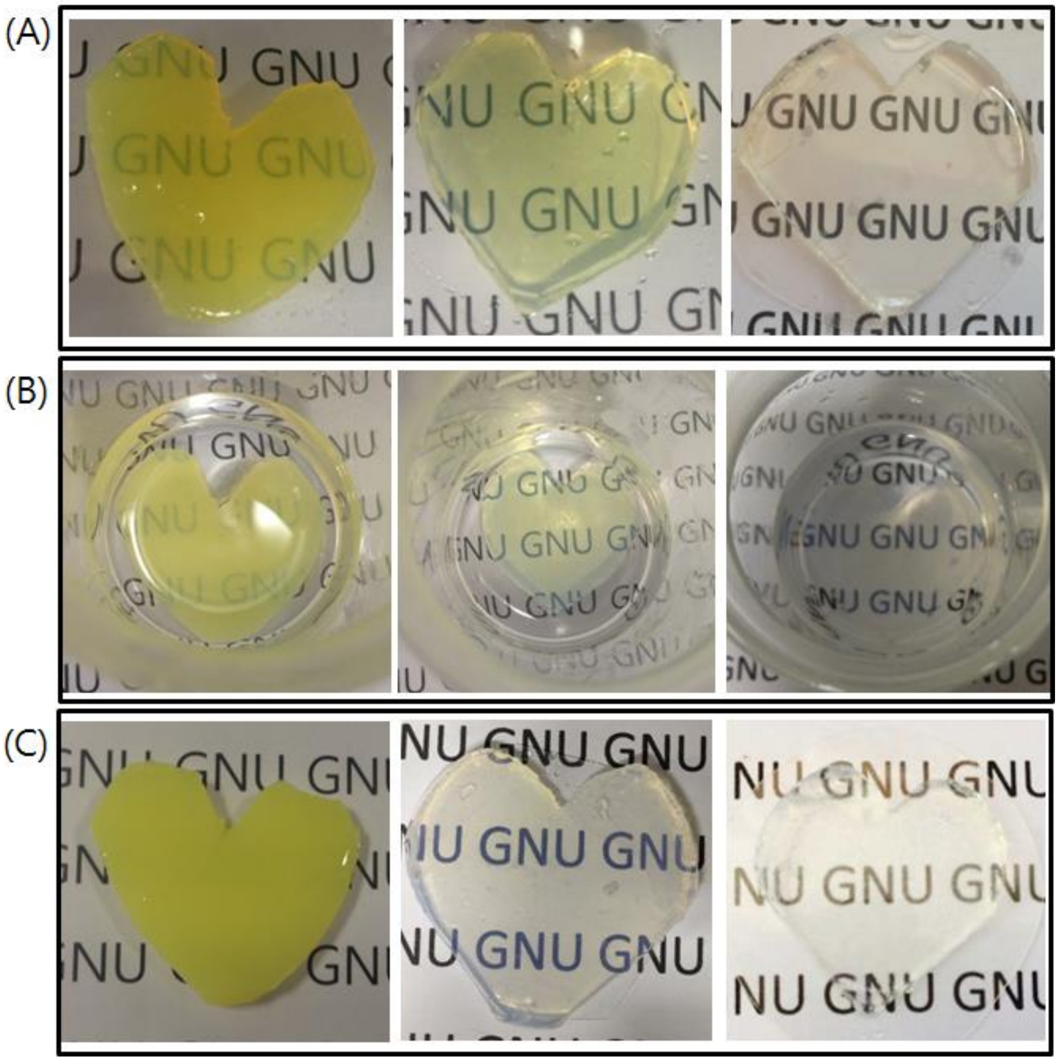

Supplementary Figure 12. Photographs of (A) organogels-5, -6 and -7 (from left to right prepared with 24, 24, and 36 hrs aging respectively), (B) organogels-5, -6 and -7 (from left to right) undergoing solvent exchange in water for hydrogel formation, (C) hydrogels-5, -6 and -7 (from left to right) after 24 hr solvent exchange with H_2O from the corresponding organogels.

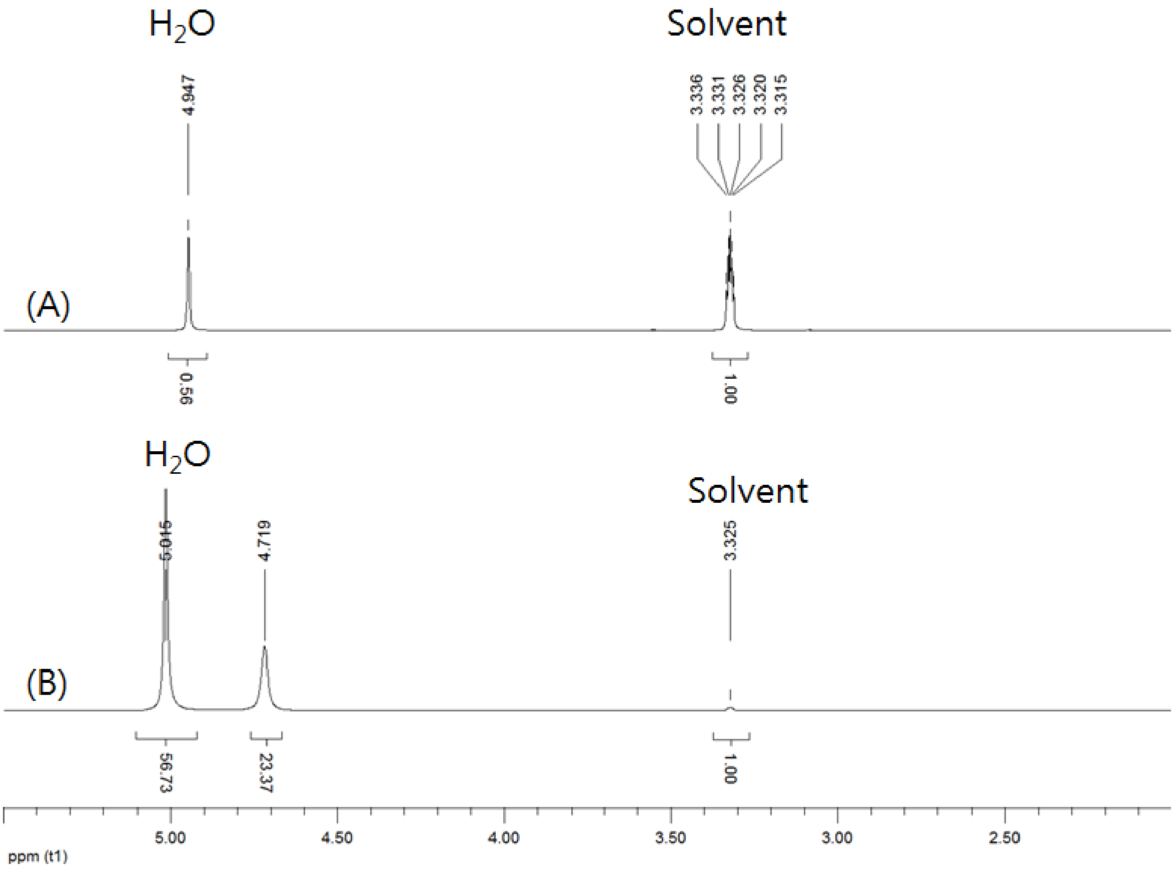

Supplementary Figure 13. NMR spectra of (A) NMR solvent as a reference sample and (B) solvent extracted from hydrogel-7 prepared with solvent exchange (NMR spectra were measured in CD_3OD). The peak at 4.719 ppm is –OH arising from the CD_3OD used as solvent.

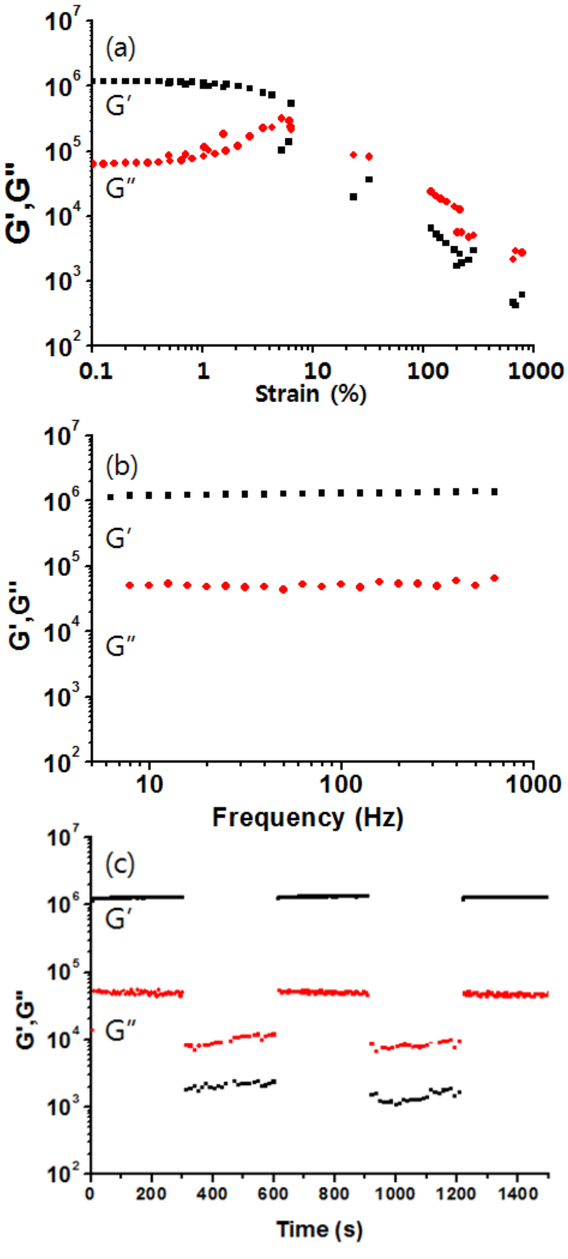

Supplementary Figure 14. Rheological properties of hydrogel-5; (a) strain sweep tests at 0.1-1000 %, (b) frequency sweep tests at 5-1000 Hz and strain 0.1 %, and (c) continuous step strain test at 0.1 % and 1000 %.

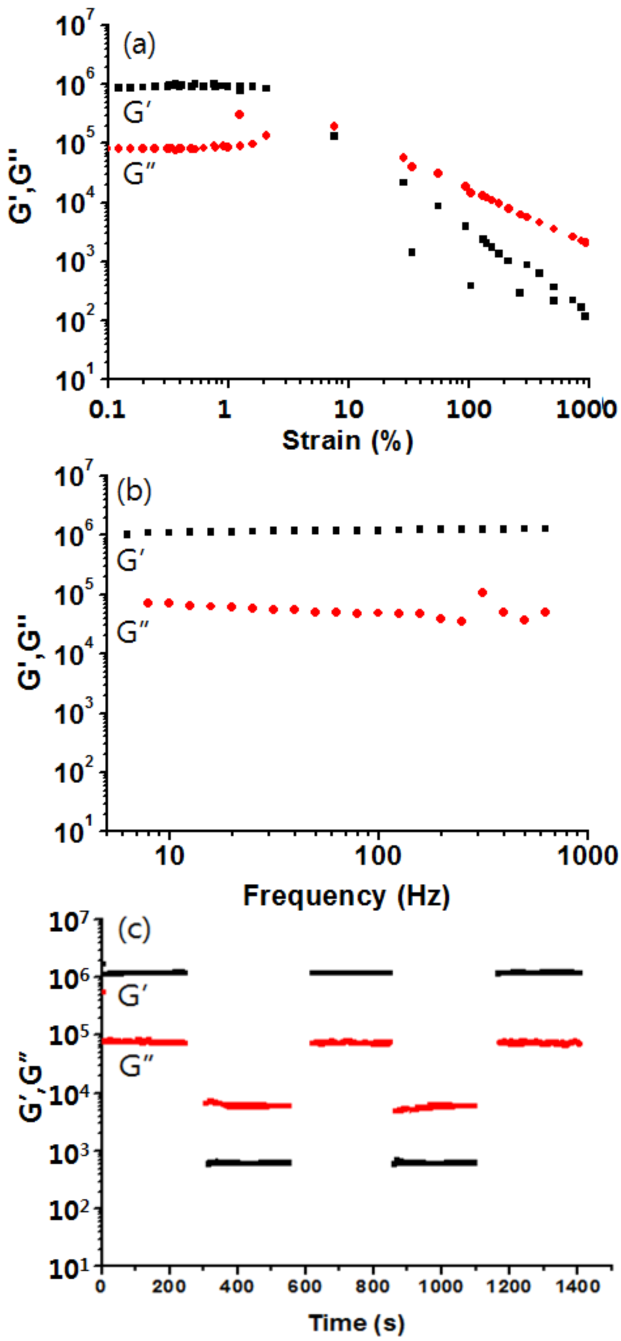

Supplementary Figure 15. Rheological properties of hydrogel-6; (a) strain sweep tests at 0.1-1000 %, (b) frequency sweep tests at 5-1000 Hz and strain 0.1 % and (c) continuous step strain test at 0.1 % and 1000 %.

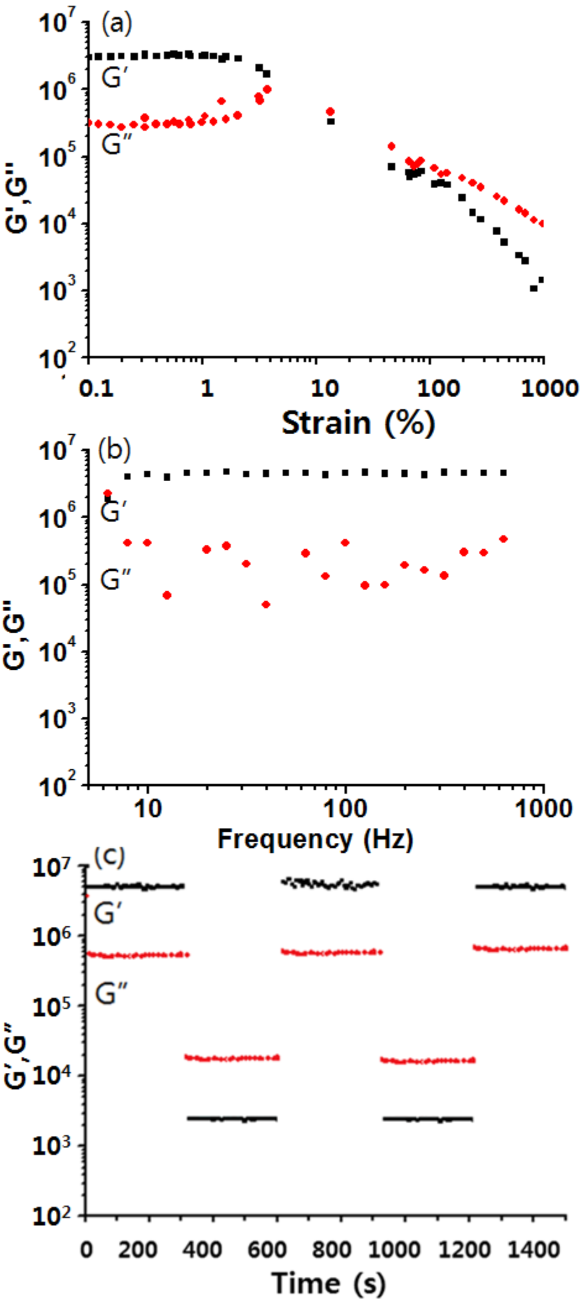

Supplementary Figure 16. Rheological properties of hydrogel-7; (a) strain sweep tests at 0.1-1000 %, (b) frequency sweep tests at 5-1000 Hz and strain 0.1 % and (c) continuous step strain test at 0.1 % and 1000 %.

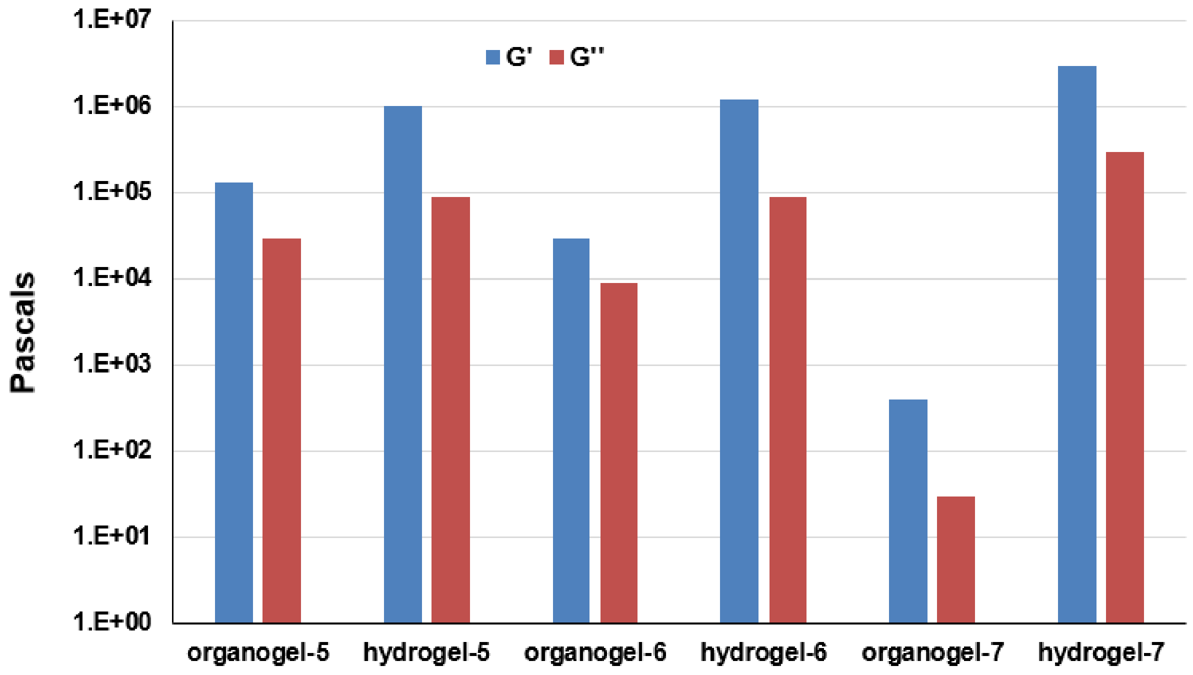

Supplementary Figure 17. Comparison of organogel and hydrogel rheological properties; the respective storage moduli G´ and loss moduli G" of the organogels measured after hydrazone formation reaction (24, 24, and 36 hr reaction for organogels-5, -6, and -7, respectively) and also the moduli after subsequent hydrogel formation achieved by immersion of corresponding organogels in water for 24 hrs (25 ^{o}C). Blue colors represent G´. Red colors represent G".

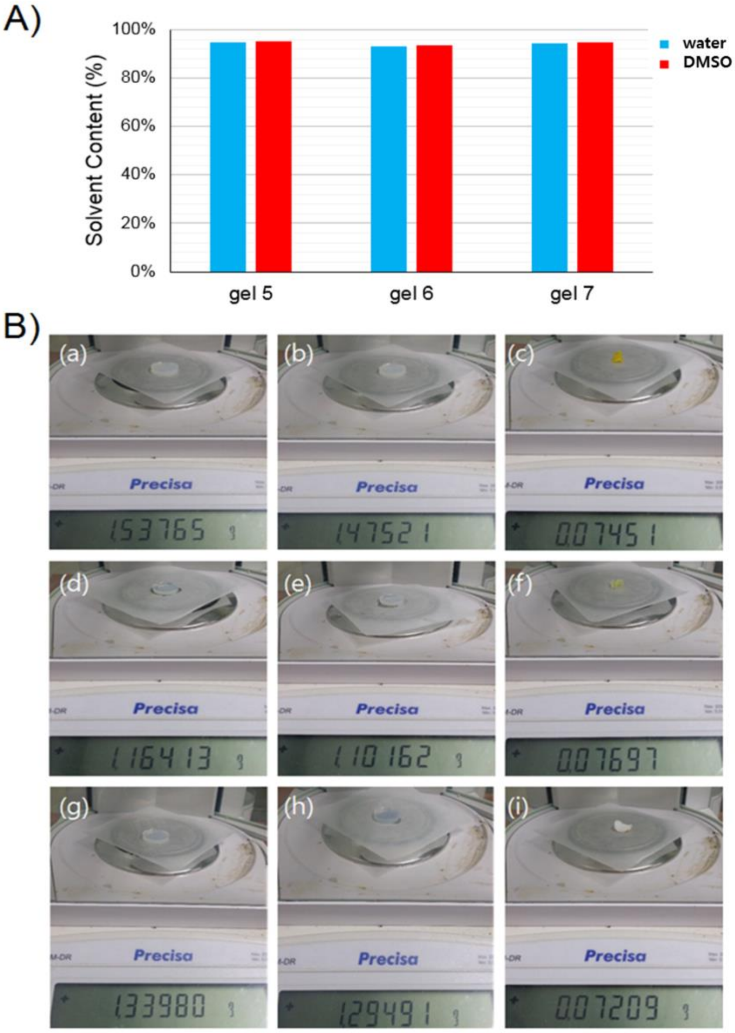

Supplementary Figure 18. (A) Comparison of solvent weight percent for water content in hydrogels (blue bars) and DMSO content in organogels (red bars) for gels prepared with 10 nmol HCl (gel 5), 5 nmol HCl (gel 6), and without HCl (gel 7). The water and similarly the DMSO contents were calculated to be between 93-95% of the gel weight from (B) the images of the weight of (a) organogel-5, (b) hydrogel-5, (c) dried gel-5, (d) organogel-6, (e) hydrogel-6, (f) dried gel-6, (g) organogel-7, (h) hydrogel-7 and (i) dried gel-7. Hydrogels (b, e and h) were prepared from organogels (a, d and g), respectively, by solvent exchange. Dried gels (c, f and i) were prepared from hydrogels (b, e and h), respectively, by vacuum drying. It is important to note that differences in sample colors occur as a result of different amount of HCl and reaction times. These organogels were prepared by 30 min, 60 min and 36 hr for organogels-5 (a), -6 (d) and -7 (g), respectively.

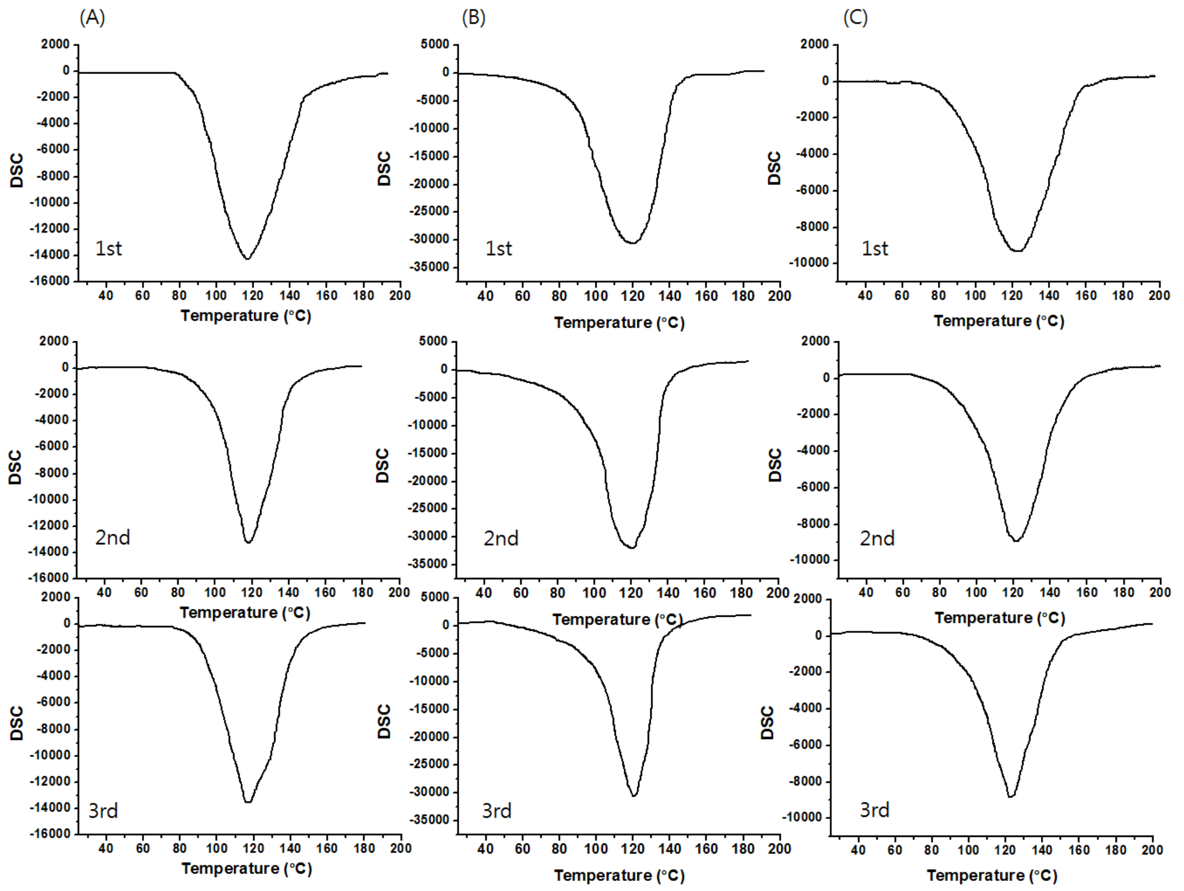

Supplementary Figure 19. Thermograms of DSC experiments for individual samples of (A) hydrogel-5, (B) hydrogel-6 and (C) hydrogel-7 prepared by 24 hr solvent exchange of organogel-5 (24 hr aging), organogel-6 (24 hr aging), and organogel-7 (36 hr aging), respectively. Each row represents subsequent measurements of the same individual samples taken after aging for an additional 24, 24, and 36 hrs for hydrogel-5, -6, and -7, respectively. To examine the gels after long aging times, each sample was examined under temperature ramping conditions rather than a temperature cycling program.

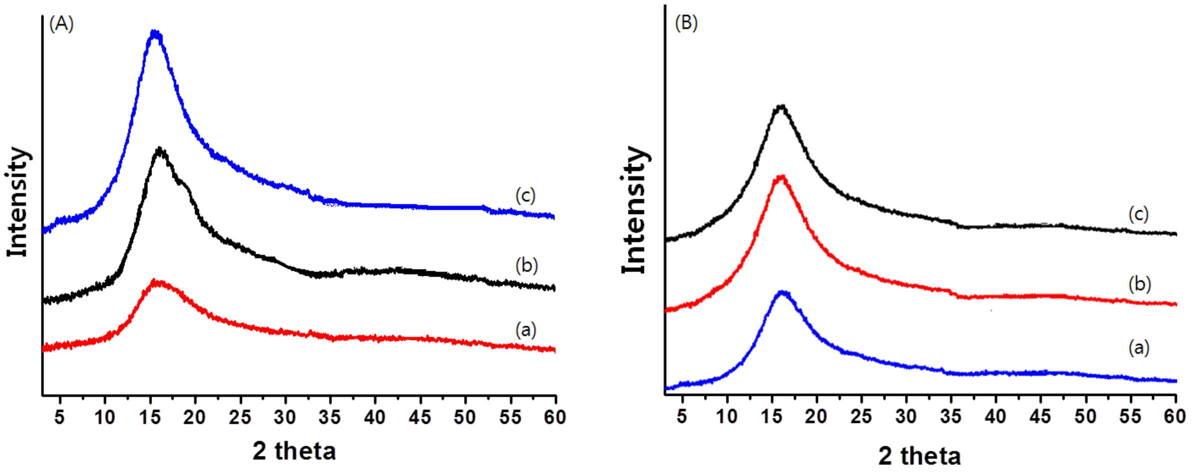

Supplementary Figure 20. Powder XRD patterns of (A) hydrogels (a) -5, (b) -6, and (c) -7 as well as (B) organogels (a) -5, (b) -6, and (c) -7 over the 2 theta range spanning 3° to 60°, which demonstrates the amorphous character of the gels.

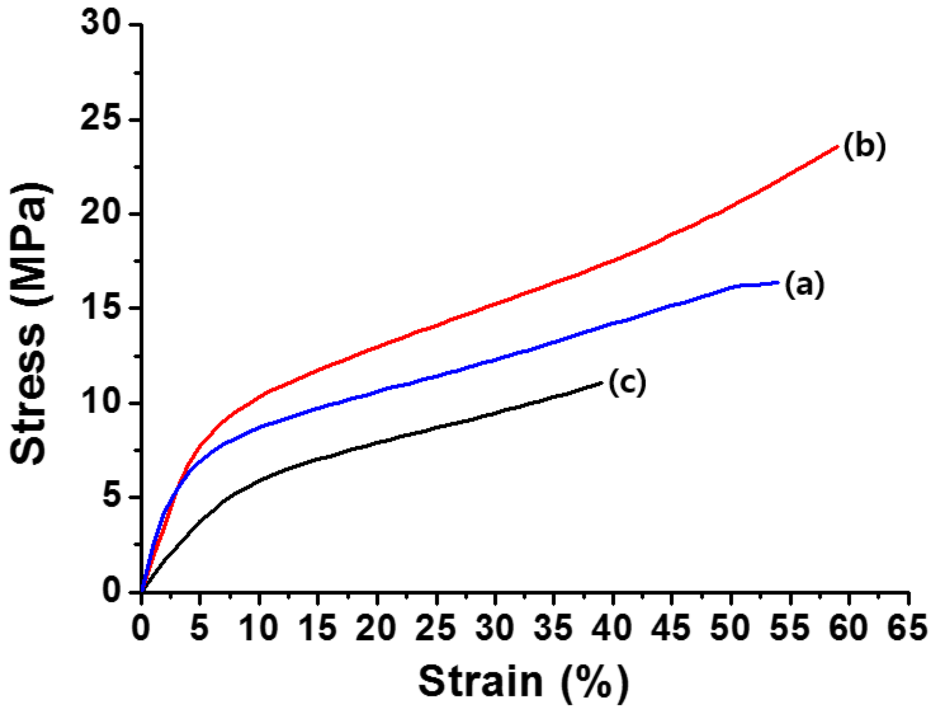

Supplementary Figure 21. The tensile stress-strain behavior of (a) hydrogel-5, (b) hydrogel-6 and (c) hydrogel-7 prepared by 24 hr solvent exchange of organogel-5 (24 hr aging), organogel-6 (24 hr aging), and organogel-7 (36 hr aging), respectively.

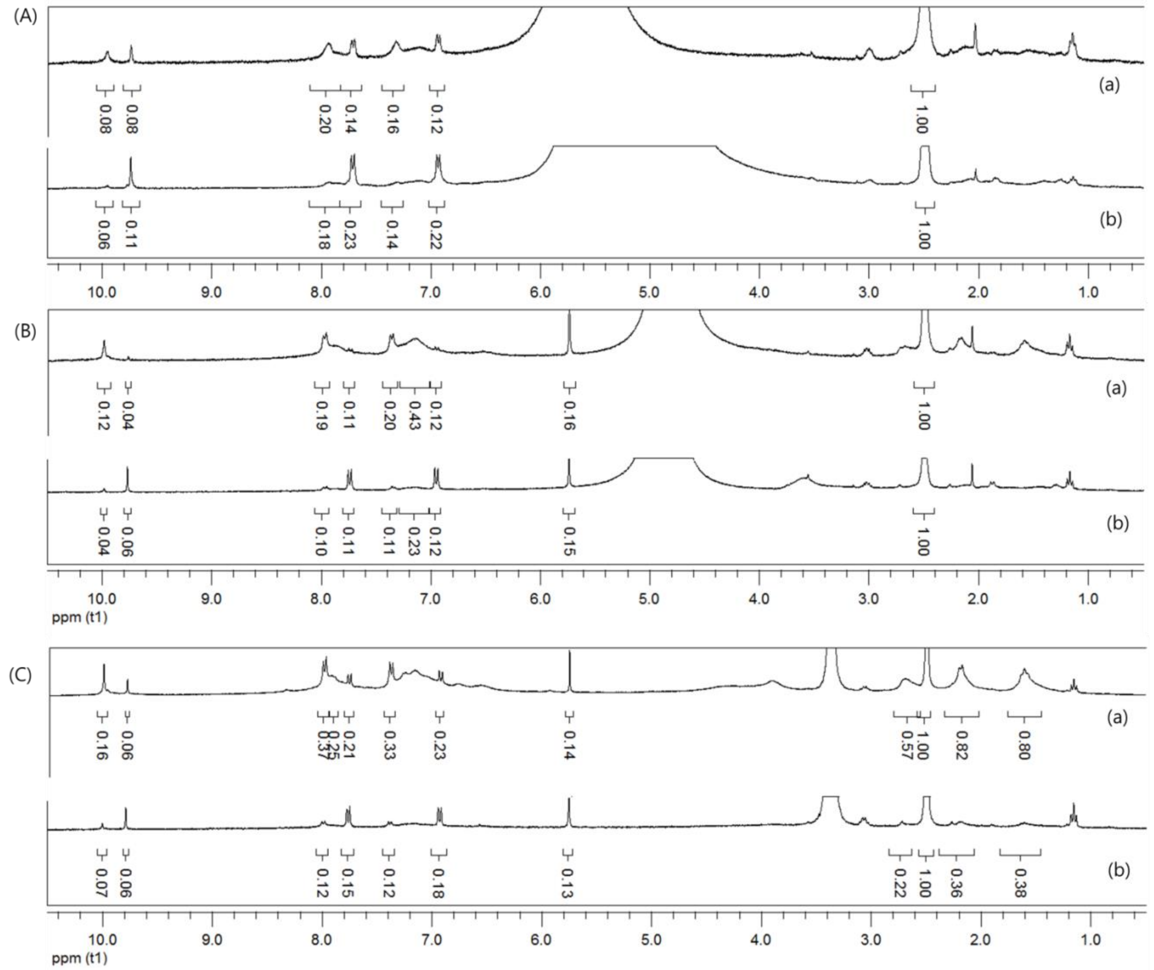

Supplementary Figure 22. NMR measurements of (A) organogel-5 (42 mM for **1** and 84 mM for **2**) (a) before and (b) after adding H_2O (20 μL). (B) organogel-6 (42 mM for **1** and 84 mM for **2**) (a) before and (b) after adding H_2O (20 μL). (C) organogel-7 (42 mM for **1** and 84 mM for **2**) (a) before and (b) after adding H_2O (20 μL).

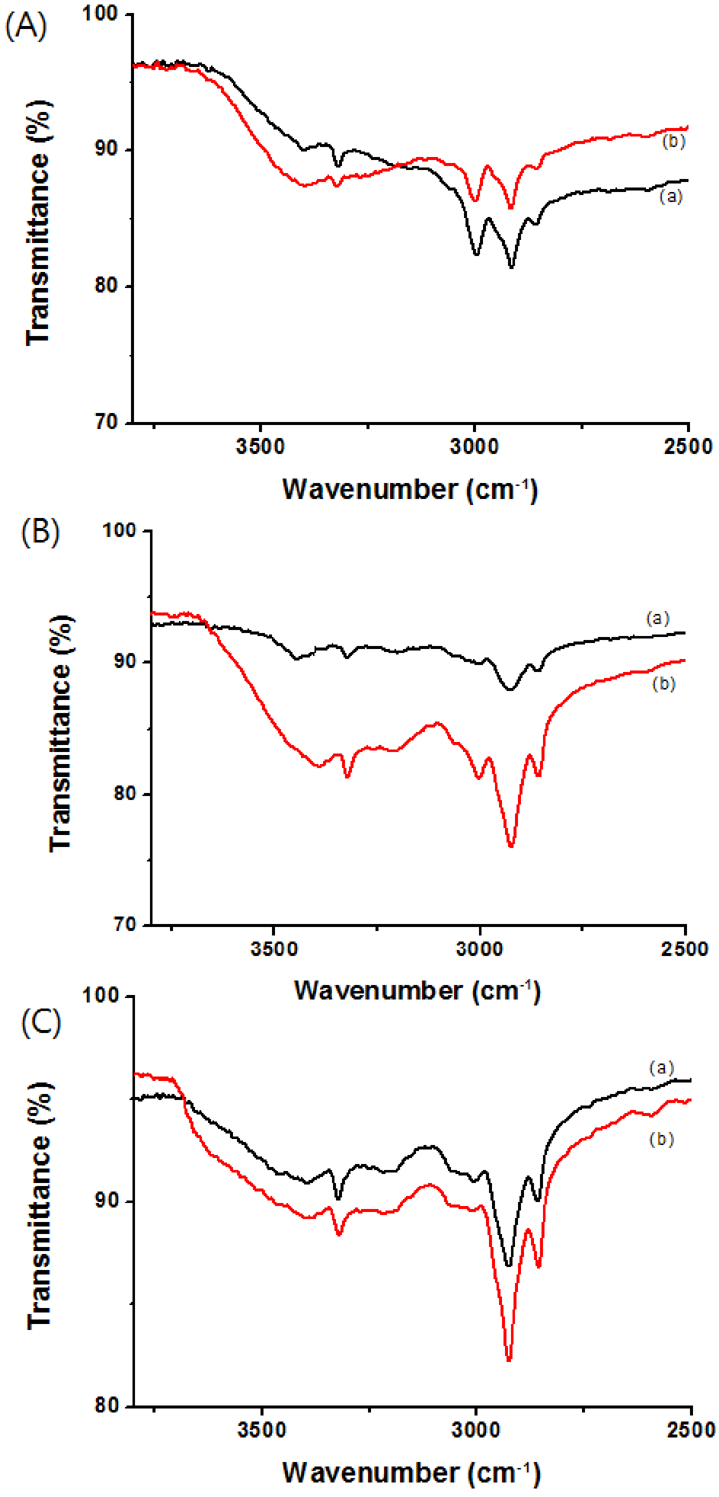

Supplementary Figure 23. IR spectra of (A) organogel-5 (black line) and hydrogel-5 (red line), (B) organogel-6 (black line) and hydrogel-6 (red line) and (C) organogel-7 (black line) and hydrogel-7 (red line). The IR spectra were obtained from freeze-dried xerogels of the respective samples indicated above.

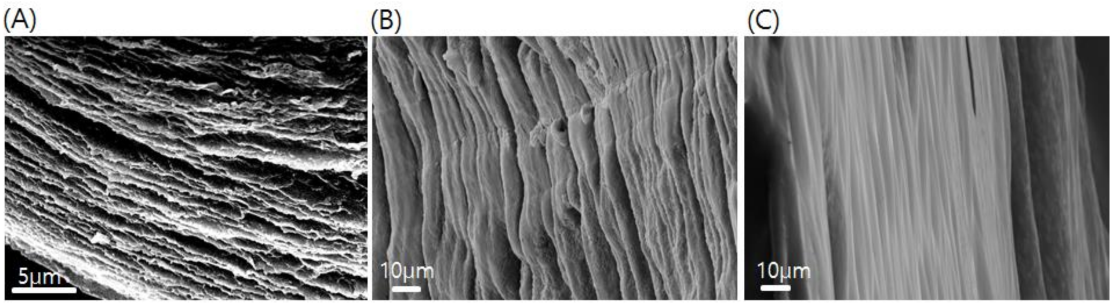

Supplementary Figure 24. SEM images of (A) hydrogel-5, (B) hydrogel-6 and (C) hydrogel-7.

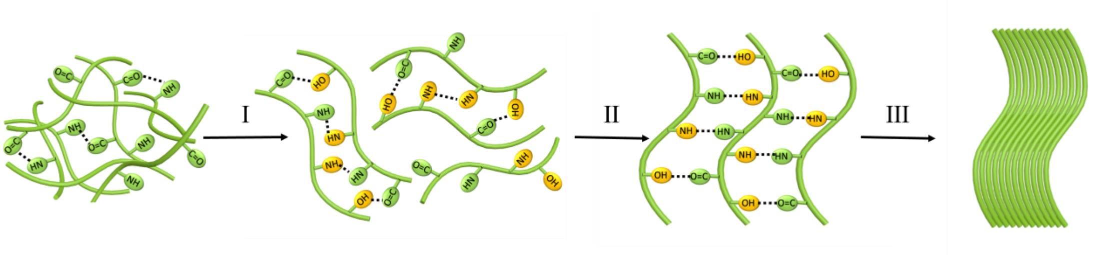

Supplementary Figure 25. Schematic representation of the morphology change during organogel to hydrogel transformation by intermolecular hydrogen-bonding interactions. As compared to the organogel fiber structure, the emergence of –OH groups and additional –NH groups provided further hydrogen-bonding sites in the hydrogel, thereby allowing extensive intermolecular hydrogen-bonding interactions in step II and III for formation into lamellar structures.

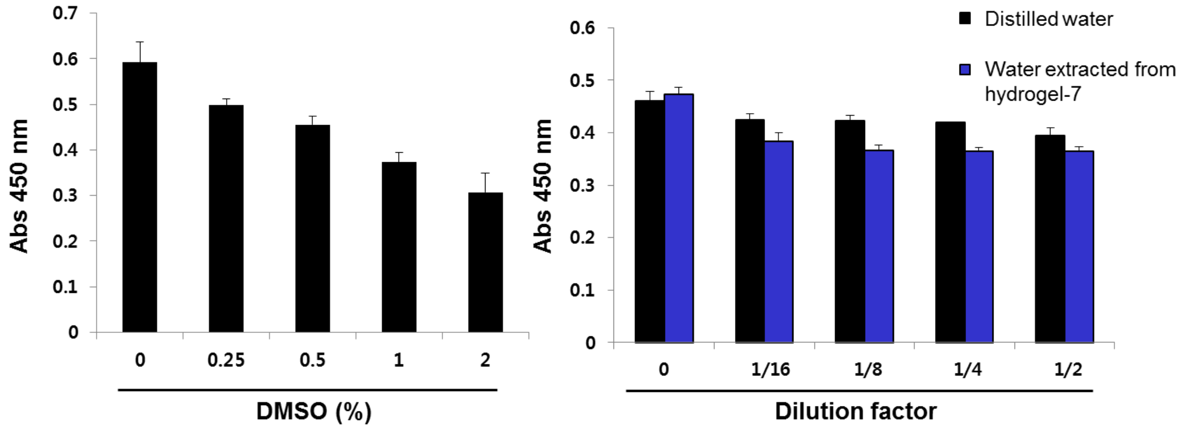

Supplementary Figure 26. Cytotoxicity assessment of hydrogel-7 as determined by MTT assay using HeLa cells (human cancer cell line).

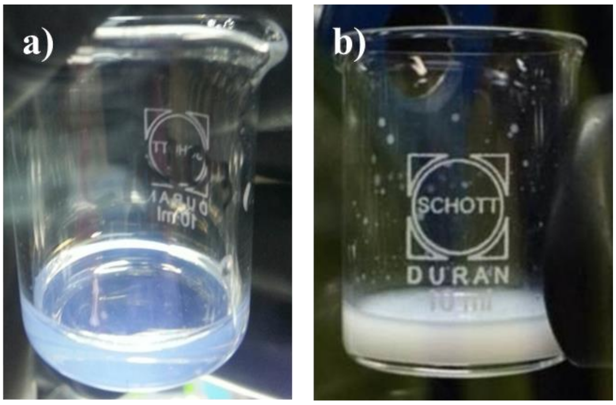

Supplementary Figure 27. Photographs of the prepared gel electrolytes: a) composite-I and b) composite-II.

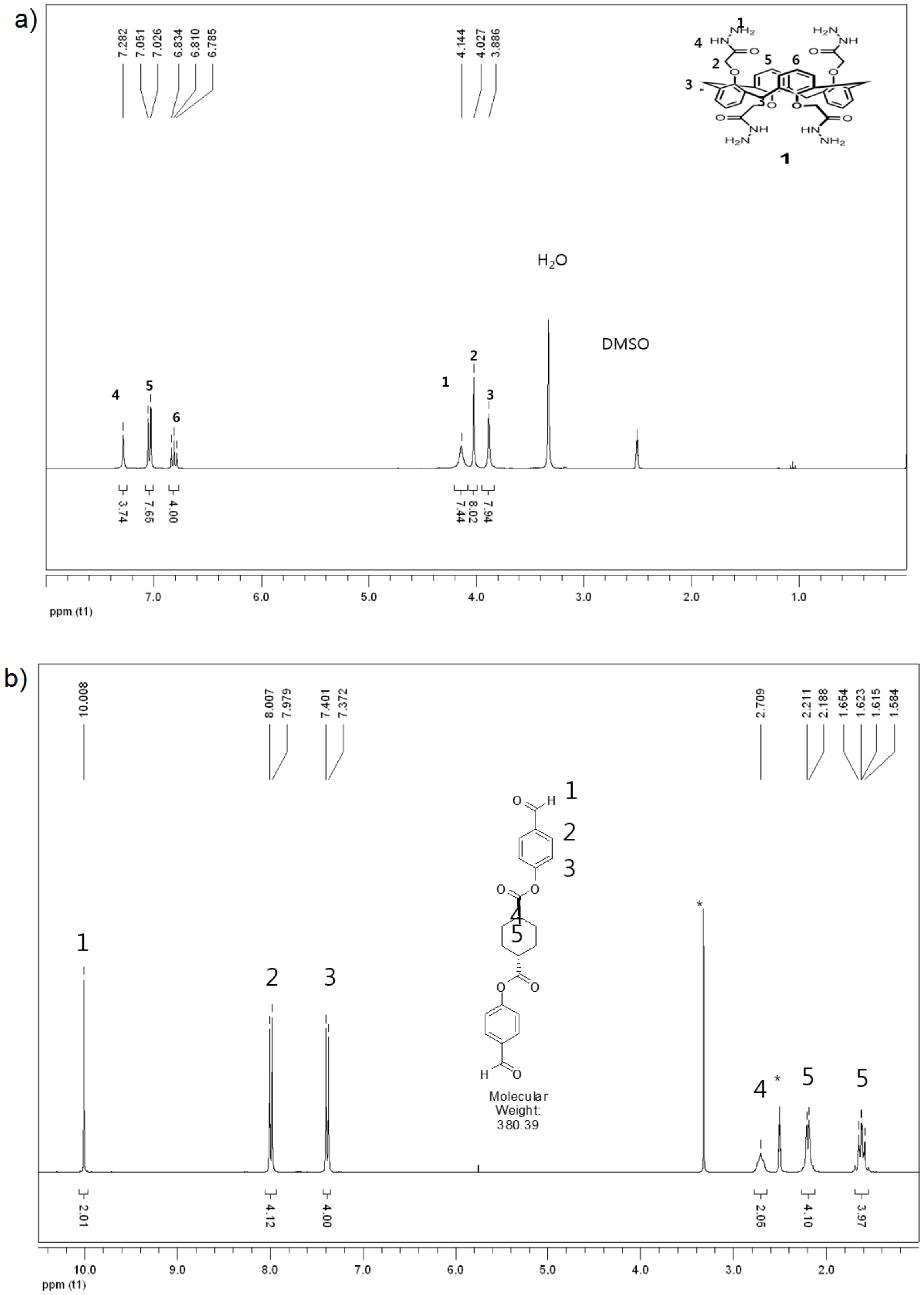

Supplementary Figure 28. ^{1}H-NMR spectra of a) compound **1** and b) compound **2**.

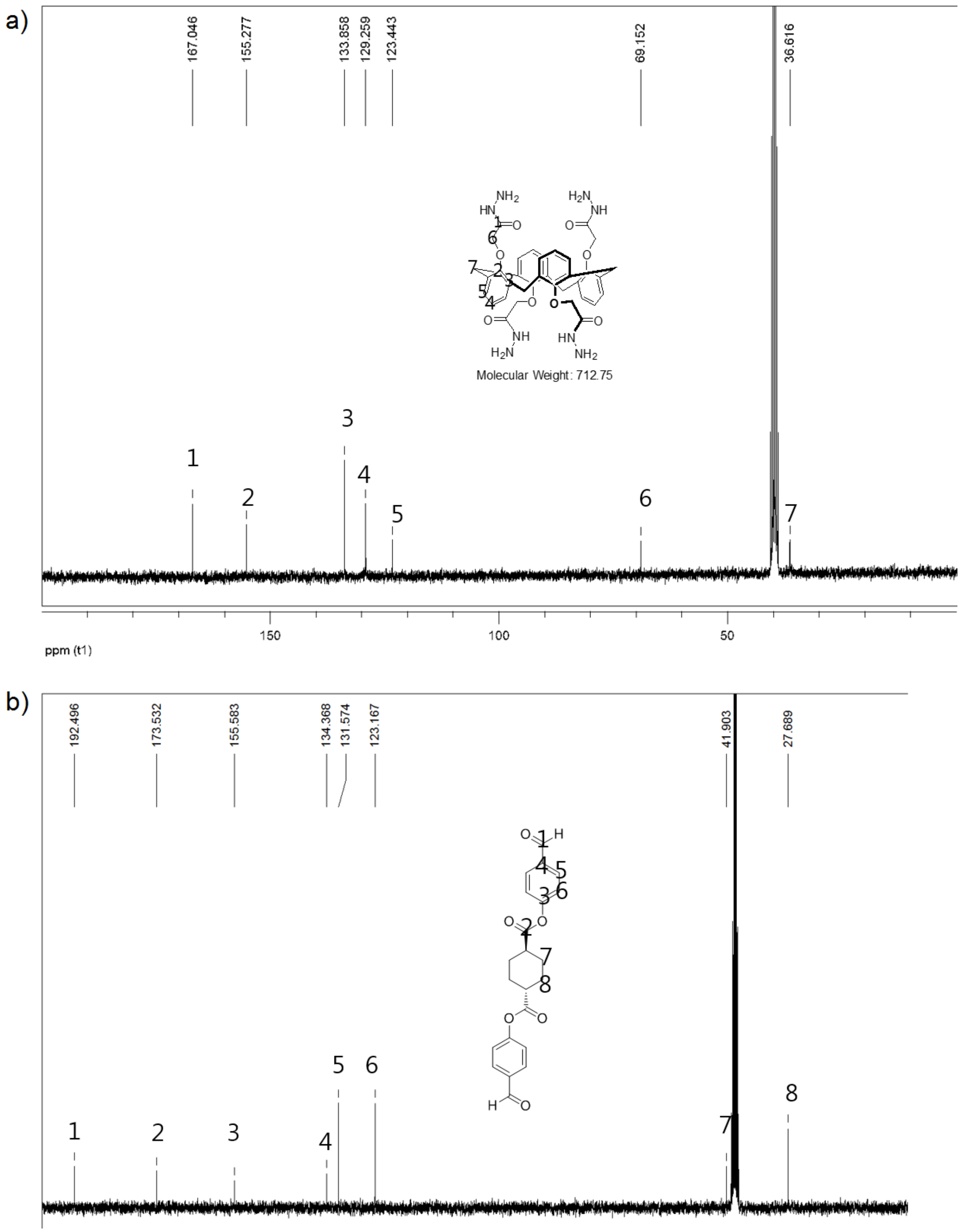

Supplementary Figure 29. ^{13}C-NMR spectra of a) compound **1** and b) compound **2**.

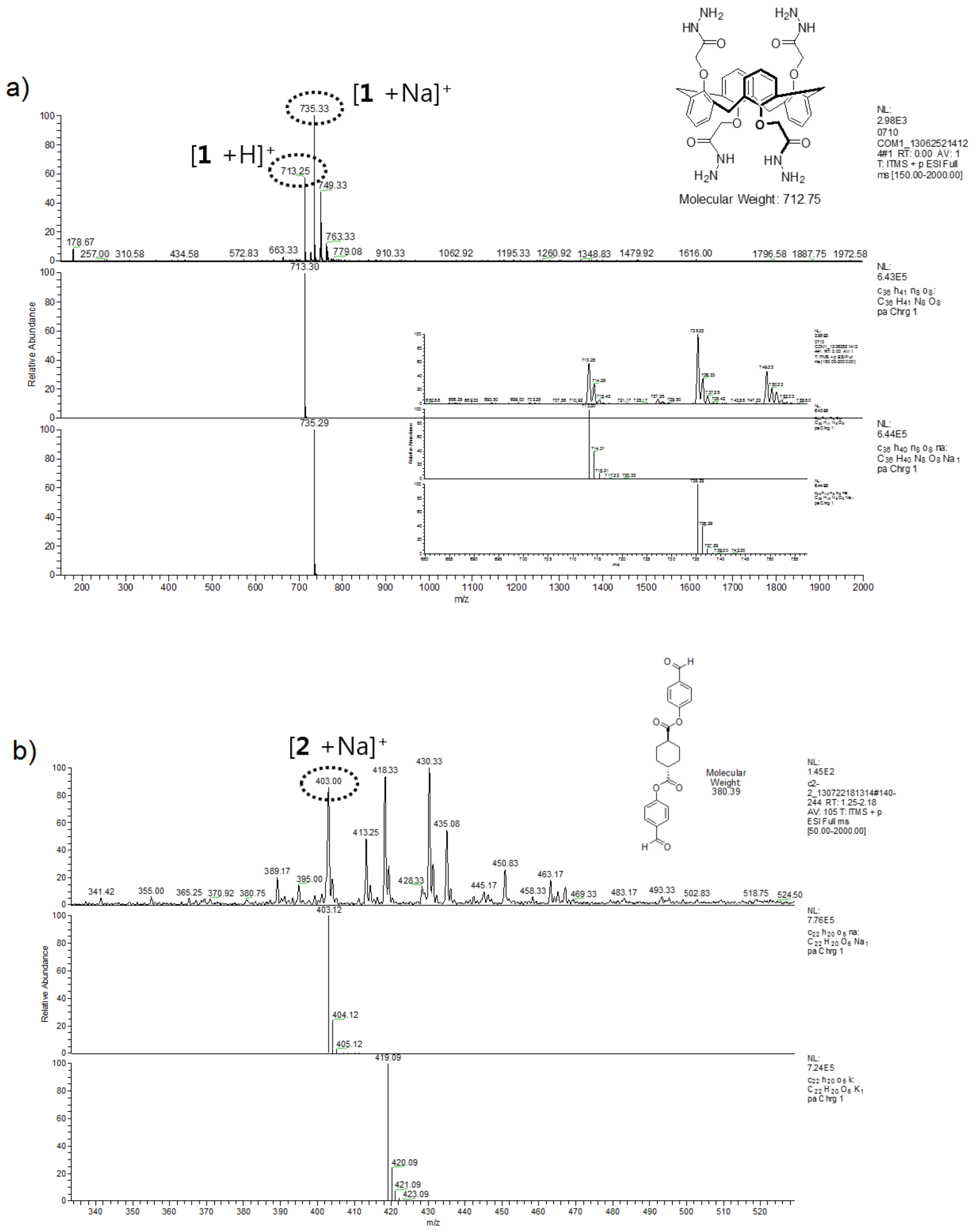

Supplementary Figure 30. Electrospray ionization mass spectra of a) compound **1** and b) **2**.

Supplementary Methods

Synthesis of 3

p-tert-Butyl phenol (150 g, 1 mol) and NaOH (1.8 g, 45 mmol) was dissolved in 37% formaldehyde (100.7 g, 1.24 mol). The reaction mixture was refluxed at 120 °C for 12 h. After the solution was cooled to room temperature, H_2O was removed *in vacuo*, and then to it was added diphenyl ether (450 mL) and toluene (150 mL). The reaction mixture was refluxed at 250 °C, again. The color of the reaction mixture changed to dark brown. Then, the crude product was recrystallized from ethyl acetate (300 mL) and was washed with acetic acid (100 mL) to give the white crystalline solid **3** in 57% yield. mp. 343 °C; IR (KBr pellet): 3176, 2958, 2866, 1603, 1482, 1361, 1242, 1200, 1042, 871, 816, 783; ^{1}H NMR (300 MHz, DMSO-d_6) δ ppm 10.36 (s, 4H), 7.07 (s, 8H), 3.52 (s, 4H), 1.23 (s, 36H); ^{13}C NMR (75 MHz DMSO-d_6) δ ppm 147.62, 143.63, 126.96, 125.74, 34.52, 33.1, 31.2; ESI-MS: Calculated for $C_{44}H_{56}O_4$ $[M+H]^+$ 649.42, Found 649.35; Anal. Calcd for $C_{44}H_{56}O_4$: C, 81.44; H, 8.70. Found: C, 81.75; H, 8.65.

Synthesis of 4

A suspension of $AlCl_3$ (24 g, 180 mmol) and toluene (150 mL) was stirred in a 1 L three-necked round-bottom flask. The contents of the flask were poured into a suspension of compound **3** (20 g, 30.8 mmol), CH_2Cl_2 (200mL), and toluene (50 mL). After the reaction mixture was stirred for 0.5 h, CH_2Cl_2 (100 mL) and 10% aqueous HCl (400 mL) solution was added to the reaction mixture in an ice bath. Finally, the reaction mixture was extracted with CH_2Cl_2 (3 x 200 mL), washed twice with water, dried over anhydrous $MgSO_4$, and the solvent was removed *in vacuo*. The crude product was recrystallized from CH_2Cl_2/ethyl ether

(1:30, v/v) to give a beige crystalline solid **4** in 67% yield (8.7 g). mp. 315 °C; IR (KBr pellet): 3160, 2935, 2870, 1594, 1465, 1448, 1244, 752; ^{1}H NMR (300 MHz, DMSO-d_6) δ ppm 9.76 (br, 4H), 7.16 (d, 8H), 6.66 (t, 4H), 3.89 (s, 4H); ^{13}C NMR (75 MHz DMSO-d_6) δ ppm 149.8, 129.2, 129.0, 121.7, 31.1; ESI-MS: Calculated for $C_{28}H_{24}O_4$ $[M+H]^+$ 425.17, Found 425.28; Anal. Calcd for $C_{28}H_{24}O_4$: C, 79.22; H, 5.70. Found: C, 79.25; H, 5.67.

Synthesis of 5

Compound **4** (5.00 g, 11.78 mmol) and Cs_2CO_3 (38.4 g, 11.78 mmol) were suspended in dry acetone (250 mL) and added to the solution of ethyl 2-bromoacetate (11.81 g, 70.68 mmol) in dry acetone (25 mL). The reaction mixture was refluxed for an additional 4 h. After cooling to room temperature, the salt was filtered and the solvent (acetone) was removed *in vacuo*. To the resulting pale yellow oil, 10% aqueous HCl (100 mL) solution and CH_2Cl_2 (100 mL) were added and the organic layer was separated and washed twice with water, dried over anhydrous $MgSO_4$, and the solvent was removed *in vacuo*. The crude product was recrystallized from CH_2Cl_2/*n*-hexane (1:30, v/v) to give a white crystalline solid **5** in 46% yield (4.12 g). mp. 118 °C; IR (KBr pellet): 3062, 2980, 2938, 1758, 1453, 1180, 1095, 1060, 769; ^{1}H NMR (300 MHz, DMSO-d_6) δ ppm 7.07 (d, 8H), 6.65 (t, 4H), 4.17 (q, 8H), 3.96 (s, 8H), 3.79 (s, 8H), 1.23 (t, 12H); ^{13}C NMR (75 MHz DMSO-d_6) δ ppm 169.9, 158.2, 133.8, 130.6, 122.7, 69.8, 60.7, 35.7, 14.5; ESI-MS: Calculated for $C_{44}H_{48}O_{12}$ $[M+Na]^+$ 791.30, Found 791.25; Anal. Calcd for $C_{44}H_{48}O_{12}$: C, 68.74; H, 6.29. Found: C, 68.72; H, 6.30.

Synthesis of 1

A solution of compound **5** (3.5 g, 4.5 mmol) in EtOH (80 mL) was refluxed for 12 h at room

temperature. The hydrazine monohydrate (4.5 g, 90 mmol) were added to the reaction mixture and refluxed for 12 h. The organic solvents were removed *in vacuo.* The crude product was recrystallized in MeOH/ethyl ether (1:1, v/v) to give a withe solid **1** as a product in 90 % yield (2.91 g). m.p. 285°C; FT-IR (KBr) : v =3414, 3397, 3373, 3255, 3019, 2916, 1685, 1617, 1570, 1528, 1459, 1445, 1353, 1323, 1249, 1209, 1191, 1161, 1096, 1060, 1012, 976, 922, 856, 772, 691, 629, 570, 555, 513, 435; ^{1}H NMR (300 MHz, DMSO-d_6) δ ppm 7.29 (s, 4H), 7.04 (d, J = 7.55 Hz, 4H), 6.81 (t, J = 7.46 Hz, 4H), 4.14 (d, J = 3.81 Hz, 8H), 4.03 (s, 8H), 3.89 (s, 8H); ^{13}C NMR (75 MHz, DMSO-d_6) 167.04, 155.27, 133.85, 129.25, 123.44, 69.15, 36.61; ESI-MS: Calculated for $C_{36}H_{40}N_8O_8$ $[M+H]^+$ 713.30 $[M+Na]^+$ 735.29, Found 713.25, 735,33; Anal. Calcd for $C_{36}H_{40}N_8O_8$: C, 60.66; H, 5.66; N, 15.72; Found: C, 60.64; H, 5.63; N, 15.74.

Synthesis of 2

To a suspension of tran-1,4-cyclohexane-dicarboxylic acid (3 g, 17.4 mmol) in toluene (30 mL) and $SOCl_2$ (12.8 mL, 174 mmol) was refluxed for 3 h and was cooled to room temperature. The solvent and unreacted $SOCl_2$ were removed *in vacuo* and CH_2Cl_2 (10 mL) was added. Then the solution of 4-hydroxybenzaldehyde (4.26 g, 15.2mmol) in CH_2Cl_2 (20 mL) and TEA (4.0 mL) was added and stirred for 3 h. After filtration of the reaction mixture, the solvent was removed *in vacuo*. The crude product was recrystallized in CH_2Cl_2/MeOH (1:30, v/v) to give a withe solid **2** as a product in 79 % yield (5.21 g). m.p. 168 ℃; FT-IR (KBr) : v =2962, 2949 2936, 2864, 1758, 1681, 1596, 1585, 1500, 1455, 1424, 1391, 1320, 1305, 1247, 1122, 1007, 970, 921, 904, 848, 776, 664, 616, 515; ^{1}H NMR (300 MHz, DMSO-d_6) δ ppm 10.01 (s, 2H) 7.99 (d, J = 8.58 Hz, 4H), 7.39 (d, J = 8.50 Hz, 4H), 2.71 (m, 2H), 2.20 (d, J = 7.08 Hz, 4H), 1.62 (m, 4H); ^{13}C NMR (75 MHz, DMSO-d_6) 192.49, 173.53, 155.58, 134.36, 131.57, 123.16, 41.90, 27.68; ESI-MS: Calculated for $C_{22}H_{20}O_6$ $[M+Na]^+$ 403.12, Found 403.12; Anal. Calcd for $C_{22}H_{20}O_6$: C, 69.46; H, 5.30. Found: C, 69.48; H, 5.31.
